# l-Type amino acid transporter 1 in hypothalamic neurons in mice maintains energy and bone homeostasis

**DOI:** 10.1172/jci.insight.154925

**Published:** 2023-04-10

**Authors:** Gyujin Park, Kazuya Fukasawa, Tetsuhiro Horie, Yusuke Masuo, Yuka Inaba, Takanori Tatsuno, Takanori Yamada, Kazuya Tokumura, Sayuki Iwahashi, Takashi Iezaki, Katsuyuki Kaneda, Yukio Kato, Yasuhito Ishigaki, Michihiro Mieda, Tomohiro Tanaka, Kazuma Ogawa, Hiroki Ochi, Shingo Sato, Yun-Bo Shi, Hiroshi Inoue, Hojoon Lee, Eiichi Hinoi

**Affiliations:** 1 Department of Bioactive Molecules, Laboratory Pharmacology, Gifu Pharmaceutical University, Gifu, Japan.; 2 Department of Neurobiology, Northwestern University, Evanston, Illinois, USA.; 3 Medical Research Institute, Kanazawa Medical University, Kahoku, Ishikawa, Japan.; 4 Faculty of Pharmacy, College of Medical, Pharmaceutical and Health, and; 5 Metabolism and Nutrition Research Unit, Institute for Frontier Science Initiative, Kanazawa University, Kanazawa, Ishikawa, Japan.; 6 Laboratory of Molecular Pharmacology, Division of Pharmaceutical Sciences, Kanazawa University Graduate School, Kanazawa, Ishikawa, Japan.; 7 Department of Integrative Neurophysiology, Graduate School of Medical Sciences, Kanazawa University, Kanazawa, Ishikawa, Japan.; 8 Department of Gastroenterology and Metabolism, Nagoya City University Graduate School of Medical Sciences, Nagoya, Aichi, Japan.; 9 Laboratory of Clinical Analytical Sciences, Division of Pharmaceutical Sciences, Kanazawa University Graduate School, Kanazawa, Ishikawa, Japan.; 10 Discovering Molecular Probes Research Unit, Institute for Frontier Science Initiative, Kanazawa University, Kanazawa, Ishikawa, Japan.; 11 Department of Rehabilitation for Motor Functions, Research Institute, National Rehabilitation Center for Persons with Disabilities, Tokorozawa, Saitama, Japan.; 12 Center for Innovative Cancer Treatment, Tokyo Medical and Dental University, Tokyo, Japan.; 13 Section on Molecular Morphogenesis, Eunice Kennedy Shriver National Institute of Child Health and Human Development, NIH, Bethesda, Maryland, USA.; 14 Department of Physiology and Metabolism, Graduate School of Medical Sciences, Kanazawa University, Kanazawa, Ishikawa, Japan.; 15 United Graduate School of Drug Discovery and Medical Information Sciences, and; 16 Division of Innovative Modality Development, Center for One Medicine Innovative Translational Research, Gifu University, Gifu, Japan.

**Keywords:** Bone Biology, Endocrinology, Amino acid metabolism, Bone disease, Obesity

## Abstract

Hypothalamic neurons regulate body homeostasis by sensing and integrating changes in the levels of key hormones and primary nutrients (amino acids, glucose, and lipids). However, the molecular mechanisms that enable hypothalamic neurons to detect primary nutrients remain elusive. Here, we identified l-type amino acid transporter 1 (LAT1) in hypothalamic leptin receptor–expressing (LepR-expressing) neurons as being important for systemic energy and bone homeostasis. We observed LAT1-dependent amino acid uptake in the hypothalamus, which was compromised in a mouse model of obesity and diabetes. Mice lacking LAT1 (encoded by solute carrier transporter 7a5, *Slc7a5*) in LepR-expressing neurons exhibited obesity-related phenotypes and higher bone mass. *Slc7a5* deficiency caused sympathetic dysfunction and leptin insensitivity in LepR-expressing neurons before obesity onset. Importantly, restoring *Slc7a5* expression selectively in LepR-expressing ventromedial hypothalamus neurons rescued energy and bone homeostasis in mice deficient for *Slc7a5* in LepR-expressing cells. Mechanistic target of rapamycin complex-1 (mTORC1) was found to be a crucial mediator of LAT1-dependent regulation of energy and bone homeostasis. These results suggest that the LAT1/mTORC1 axis in LepR-expressing neurons controls energy and bone homeostasis by fine-tuning sympathetic outflow, thus providing in vivo evidence of the implications of amino acid sensing by hypothalamic neurons in body homeostasis.

## Graphical abstract

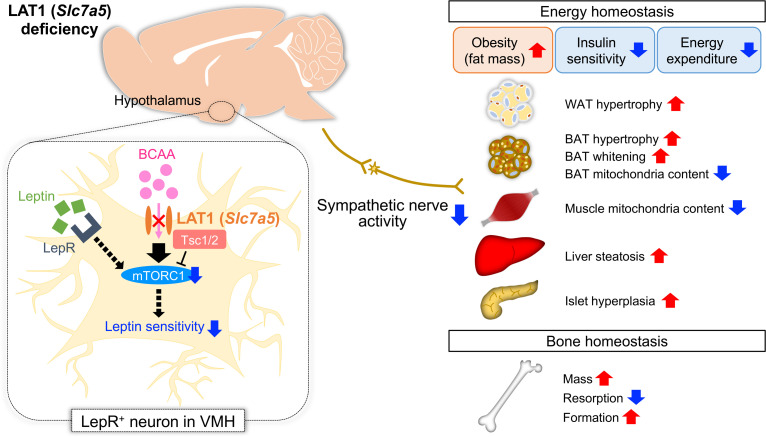

## Introduction

Hypothalamic neurons in the central nervous system (CNS) serve as pivotal regulators of body homeostasis by receiving and integrating peripheral signals (1, 2). Key hormones and pathways involved in the hypothalamic regulation of energy and bone homeostasis have been identified. For example, leptin signaling in hypothalamic neurons plays a major role in the regulation of energy homeostasis in a neuron type– and context-dependent manner via downstream effectors such as signal transducer and activator of transcription 3 (STAT3) and mechanistic target of rapamycin complex-1 (mTORC1) (3, 4). Moreover, leptin is a central regulator that inhibits bone formation partly through the sympathetic nervous system (SNS) (5). In addition to hormones, hypothalamic neurons sense primary nutrients (amino acids, glucose, and lipids) and coordinate metabolic responses (6).

Appropriate consumption of protein and amino acids is widely acknowledged as beneficial for energy homeostasis. Among the amino acids, branched-chain amino acids (BCAAs; leucine, isoleucine, and valine) largely escape first-pass metabolism, resulting in a marked increase in their levels in the brain as well as in plasma in the postprandial state (7–10). Elevated levels of circulating BCAAs have been linked to the development of obesity in both rodents and humans (11, 12). Long-term consumption of BCAA-rich diet causes hyperphagia and obesity, leading to a shortened life span (13). A recent study showed that reduced isoleucine intake is required for the metabolic benefits of a low-protein diet (14). In addition, BCAAs have been implicated in bone homeostasis. Dietary essential amino acid supplements containing leucine, isoleucine, and valine increased bone strength in ovariectomized osteoporotic rats (15). Furthermore, high leucine intake is associated with high bone mineral density (BMD) in female monozygotic twins (16), and high BCAA levels are associated with a low risk of BMD decline (17).

l-type amino acid transporter 1 (LAT1), encoded by solute carrier transporter 7a5 (*Slc7a5*), is a Na^+^-independent amino acid transporter that facilitates the cellular uptake of large neutral amino acids, including BCAAs and aromatic amino acids (18, 19), and plays a crucial role in amino acid sensing and signaling in specific cell types (20, 21), contributing to the pathogenesis of cancer and neurological disorders (22, 23). We recently reported that *Slc7a5* deficiency in osteoclasts or chondrocytes causes bone loss or spinal deformity, respectively (24, 25). Global *Slc7a5* deficiency in mice causes drastic neural defects leading to embryonic lethality (26, 27). LAT1/*Slc7a5* is expressed in the neural tube, forebrain, and postnatal neurogenic regions (27–29) and in endothelial cells of the blood-brain barrier, wherein it regulates BCAA levels in the brain (30). LAT1-dependent leucine uptake contributes to cellular homeostasis through mTORC1 signaling in specific cell types (20, 21), and leucine regulates hypothalamic activity to control energy status through mTORC1 signaling (31, 32). However, physiological and pathological functions and underlying action mechanisms of the LAT1/mTORC1 axis in hypothalamic neurons with respect to body homeostasis remain unknown.

Obesity and osteoporosis, two common chronic metabolic diseases, are worldwide health problems with significant impact on patient quality of life and with high mortality rates and health care costs (33, 34). To improve treatments for these diseases, deeper insights into energy and bone homeostasis are required. In the present study, we found that the LAT1/mTORC1 axis in hypothalamic neurons plays a pivotal role in energy and bone homeostasis as an amino acid sensor, thereby providing valuable information on a potentially novel and common target for the treatment of obesity and osteoporosis.

## Results

### Hypothalamus has a LAT1-dependent amino acid uptake system, which is suppressed in an obesity and diabetes mouse model.

**Figure 1:**
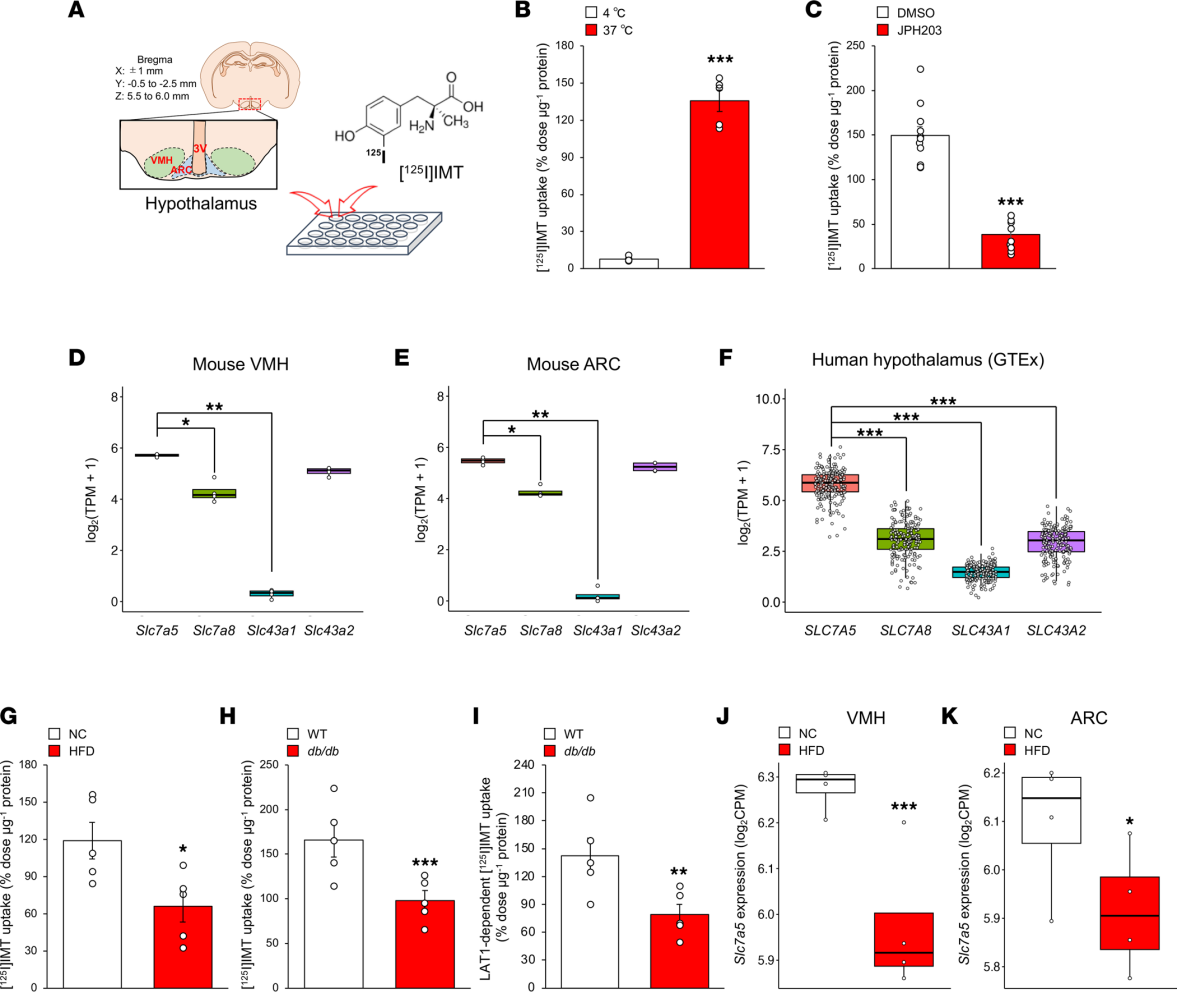
Hypothalamus has a LAT1-dependent amino acid uptake system that is altered by metabolic states. (**A**) Schematic diagram of the procedure for [^125^I]IMT uptake assay. (**B** and **C**) Brain tissues from wild-type mice at 8–12 weeks of age were incubated with [^125^I]IMT at 4°C or 37°C for 30 minutes in Hanks’ balanced salt solution (HBSS) buffer (**B**) (*n* = 5, ***P < 0.001, 2-tailed Student’s *t* test) or at 37°C for 30 minutes in HBSS buffer containing 30 μM JPH203 (**C**) (*n* = 10 or 11, ***P < 0.001, 2-tailed Student’s *t* test). (**D** and **E**) Quantification of mRNAs encoding LAT family in VMH (**D**) and ARC (**E**) of mice fed a normal chow diet (NC) at 8–12 weeks of age (*n* = 4, *P < 0.05, **P < 0.01, Kruskal-Wallis test post hoc Dunn’s test). TPM, transcripts per million. (**F**) Quantification of mRNAs encoding LAT family in human hypothalamus (*n* = 202, ***P < 0.001, Kruskal-Wallis test post hoc Dunn’s test). (**G**) Brain tissues from WT mice fed an NC or HFD (for 17 weeks, from 7 to 24 weeks of age) at 24 weeks of age were incubated with [^125^I]IMT at 37°C for 30 minutes in HBSS buffer (*n* = 5, *P < 0.05, 2-tailed Student’s *t* test). (**H**) Brain tissues from WT mice and *db/db* mice at 24 weeks of age were incubated with [^125^I]IMT at 37°C for 30 minutes in HBSS buffer (*n* = 5, ***P < 0.001, 2-tailed Student’s *t* test). (**I**) LAT1-dependent [^125^I]IMT uptake in brain tissues from WT mice and *db/db* mice at 24 weeks of age (*n* = 5, **P < 0.01, 2-tailed Student’s *t* test). (**J** and **K**) Quantification of *Slc7a5* mRNA in VMH (**J**) and ARC (**K**) of mice fed an NC or HFD (from 5–6 to 8–12 weeks of age) at 8–12 weeks of age (*n* = 4, *P < 0.05, ***P < 0.001, Fisher’s exact test). All the mice used in this study were male. CPM, counts per million.

We first assessed whether a LAT-dependent amino acid transport system was operational in the hypothalamus of mice. To this end, we synthesized l-3-[^125^I]iodo-α-methyltyrosine ([^125^I]IMT) and tested whether microdissected mouse hypothalami took up [^125^I]IMT, since its accumulation is mainly mediated by LATs (35) (Figure 1A). [^125^I]IMT uptake was temperature dependent and significantly reduced by JPH203, a specific inhibitor of LAT1, in the hypothalamus (Figure 1, B and C).

The LAT family, which consists of LAT1 (*SLC7A5*), LAT2 (*SLC7A8*), LAT3 (*SLC43A1*), and LAT4 (*SLC43A2*), transports neutral amino acids into cells with possibly redundant functions (22). Analysis of an RNA-Seq data set revealed that *Slc7a5* expression was the highest among the LAT family in both the ventromedial hypothalamus (VMH) and arcuate nucleus (ARC) of the murine hypothalamus (Figure 1, D and E). Consistently, *SLC7A5* expression was the highest among the LAT family in the human hypothalamus according to the Genotype-Tissue Expression (GTEx) database (Figure 1F).

We next examined whether metabolic states affected LAT1-dependent amino acid uptake. [^125^I]IMT uptake was significantly decreased in the hypothalamus of both high-fat diet–fed (HFD-fed) mice and *db/db* mice (Figure 1, G and H). Moreover, JPH203-sensitive (LAT1-dependent) [^125^I]IMT uptake was significantly decreased in *db*/*db* mice (Figure 1I). In addition, *Slc7a5* expression was significantly downregulated by HFD exposure in both the VMH and ARC in the murine hypothalamus (Figure 1, J and K).

Collectively, these results demonstrate that the hypothalamus has a LAT1-dependent amino acid uptake system that can be altered by metabolic states such as obesity and diabetes.

### LAT1 in LepR-expressing neurons is necessary for maintenance of body weight and amino acid level balance.

**Figure 2:**
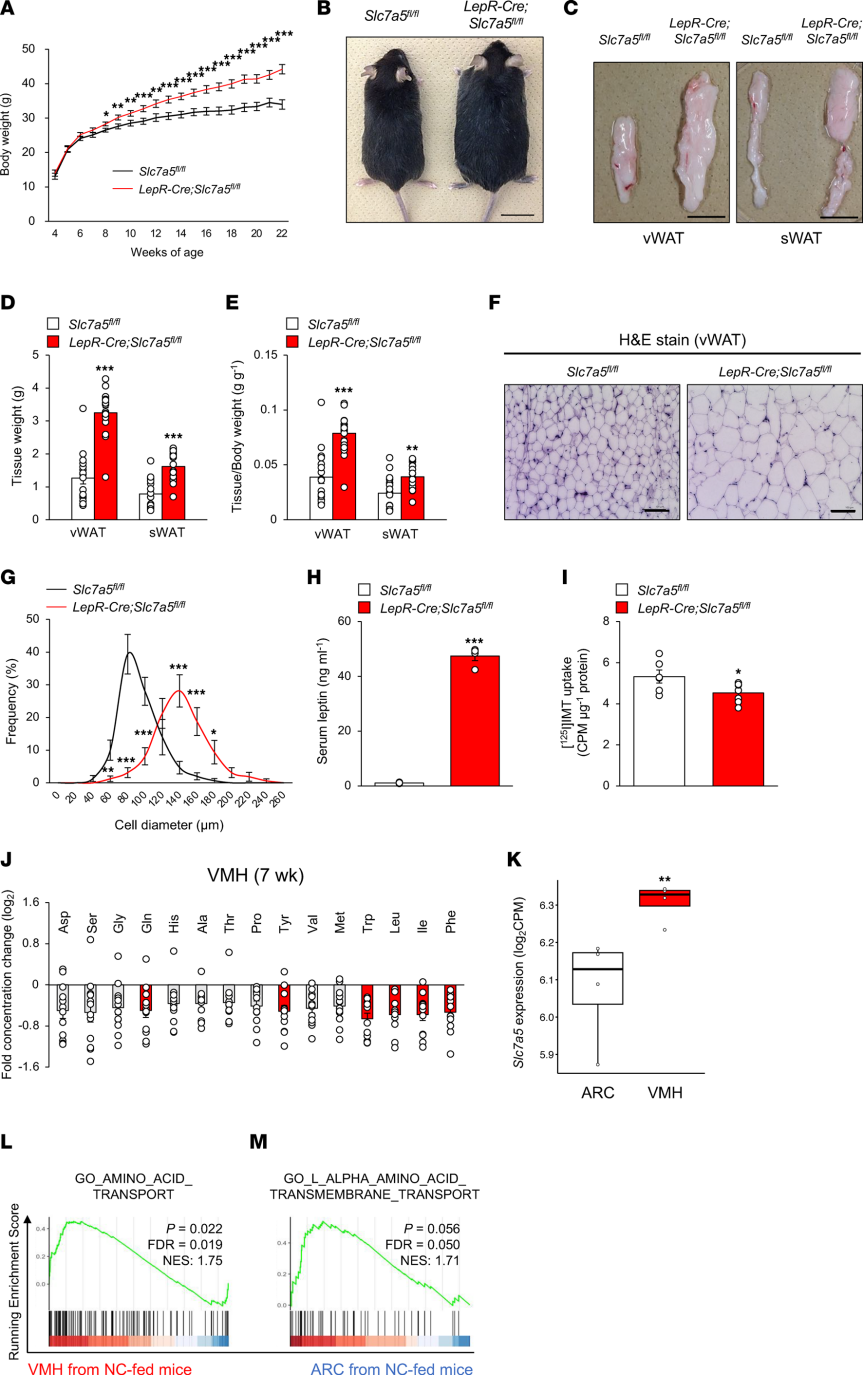
LAT1 in LepR-expressing neurons plays a critical role in the maintenance of proper body weight and adequate amino acid level balance. (**A**) Weekly body weight is shown for *LepR-Cre Slc7a5^fl/fl^* mice and control mice fed an NC (*n* = 8 or 9, *P < 0.05, **P < 0.01, ***P < 0.001, 2-way ANOVA with Bonferroni post hoc test). (**B**) Gross appearance at 24 weeks of age is shown for *LepR-Cre Slc7a5^fl/fl^* mice and control mice fed an NC. Scale bar, 1 cm. (**C**–**E**) Representative pictures of visceral and subcutaneous WAT (vWAT and sWAT) (**C**), adipose tissue weights (**D**), and adipose tissue weights normalized to body weight (**E**) are shown for *LepR-Cre Slc7a5^fl/fl^* mice and control mice at 22–24 weeks of age (*n* = 17 or 18, **P < 0.01, ***P < 0.001, 2-tailed Student’s *t* test). Scale bar, 1 cm. (**F** and **G**) H&E stain was performed on the vWAT of *LepR-Cre Slc7a5^fl/fl^* mice and control mice at 22–24 weeks of age, followed by quantification of adipocyte size of vWAT (*n* = 15 or 16, *P < 0.05, **P < 0.01, ***P < 0.001, 2-way ANOVA with Bonferroni post hoc test). Scale bar, 100 μm. (**H**) Serum leptin levels were measured in *LepR-Cre Slc7a5^fl/fl^* mice at 22–24 weeks of age (*n* = 4, ***P < 0.001, 2-tailed Student’s *t* test). (**I**) [^125^I]IMT uptake in hypothalamus from *LepR-Cre Slc7a5^fl/fl^* mice at 22–24 weeks of age (*n* = 6 or 7, *P < 0.05, 2-tailed Student’s *t* test). (**J**) Log_2_ ratio of the amino acid levels in VMH between *LepR-Cre Slc7a5^fl/fl^* mice and control mice at 7 weeks of age (*n* = 12, the amino acids with *P* < 0.05 are represented in red, 2-tailed Student’s *t* test). (**K**) Quantification of *Slc7a5* mRNA in ARC and VMH of mice fed an NC (*n* = 4, **P < 0.01, Fisher’s exact test). (**L** and **M**) The enrichment plots for amino acid transport-related gene sets in VMH and ARC of mice fed an NC (*n* = 4). All the mice used in this study were male.

To determine the physiological importance of LAT1 in leptin receptor–expressing (LepR-expressing) neurons in systemic energy homeostasis, we generated *LepR-Cre Slc7a5^fl/fl^* mice by crossing *Slc7a5^fl/fl^* mice with *LepR-Cre* mice. *LepR-Cre* mice did not exhibit any abnormalities in body weight, glucose tolerance, insulin sensitivity, and adipose tissue weights (Supplemental Figure 1, A–E; supplemental material available online with this article; https://doi.org/10.1172/jci.insight.154925DS1). We confirmed the deletion efficiency and specificity in *LepR-Cre Slc7a5^fl/fl^* mice (Supplemental Figure 2, A–C). The body weight of *LepR-Cre Slc7a5^fl/fl^* mice began to significantly diverge from that of control mice by 8 weeks after birth, accompanied by a larger gross appearance at 24 weeks of age (Figure 2, A and B). Moreover, visceral and subcutaneous fat pads were significantly larger in *LepR-Cre Slc7a5^fl/fl^* mice with adipocyte hypertrophy and higher circulating leptin levels (Figure 2, C–H). Brain morphology, neuronal cytoarchitecture, and the number of LepR-expressing neurons in the VMH or ARC were comparable between control and *LepR-Cre Slc7a5^fl/fl^* mice (Supplemental Figure 2, D–J).

We then assessed how the ablation of LAT1 expression in LepR-expressing cells might influence amino acid levels in the hypothalamus. [^125^I]IMT uptake was significantly decreased in the hypothalamus of *LepR-Cre Slc7a5^fl/fl^* mice before the onset of obesity (7 weeks of age) (Figure 2I). Among the amino acid substrates of LAT1, leucine, isoleucine, phenylalanine, tyrosine, and tryptophan levels were significantly decreased in the VMH of *LepR-Cre Slc7a5^fl/fl^* mice with a trend toward decreased valine levels compared with those in control mice (Figure 2J). Conversely, no significant changes were observed in amino acid levels in the ARC and plasma between control and *LepR-Cre Slc7a5^fl/fl^* mice (Supplemental Figure 2, K and L). Consistently, analysis of RNA-Seq data revealed that the *Slc7a5* mRNA level was significantly higher in the VMH than in the ARC, and gene sets involved in amino acid transport were enriched in the VMH compared with those in the ARC (Figure 2, K–M).

Collectively, these results indicate that LAT1 depletion in LepR-expressing neurons leads to spontaneous obesity owing to an increase in fat mass, concomitant with reduction in BCAA uptake in the VMH.

### LAT1 in LepR-expressing neurons is implicated in insulin sensitivity, energy homeostasis, and brown adipose tissue function.

**Figure 3:**
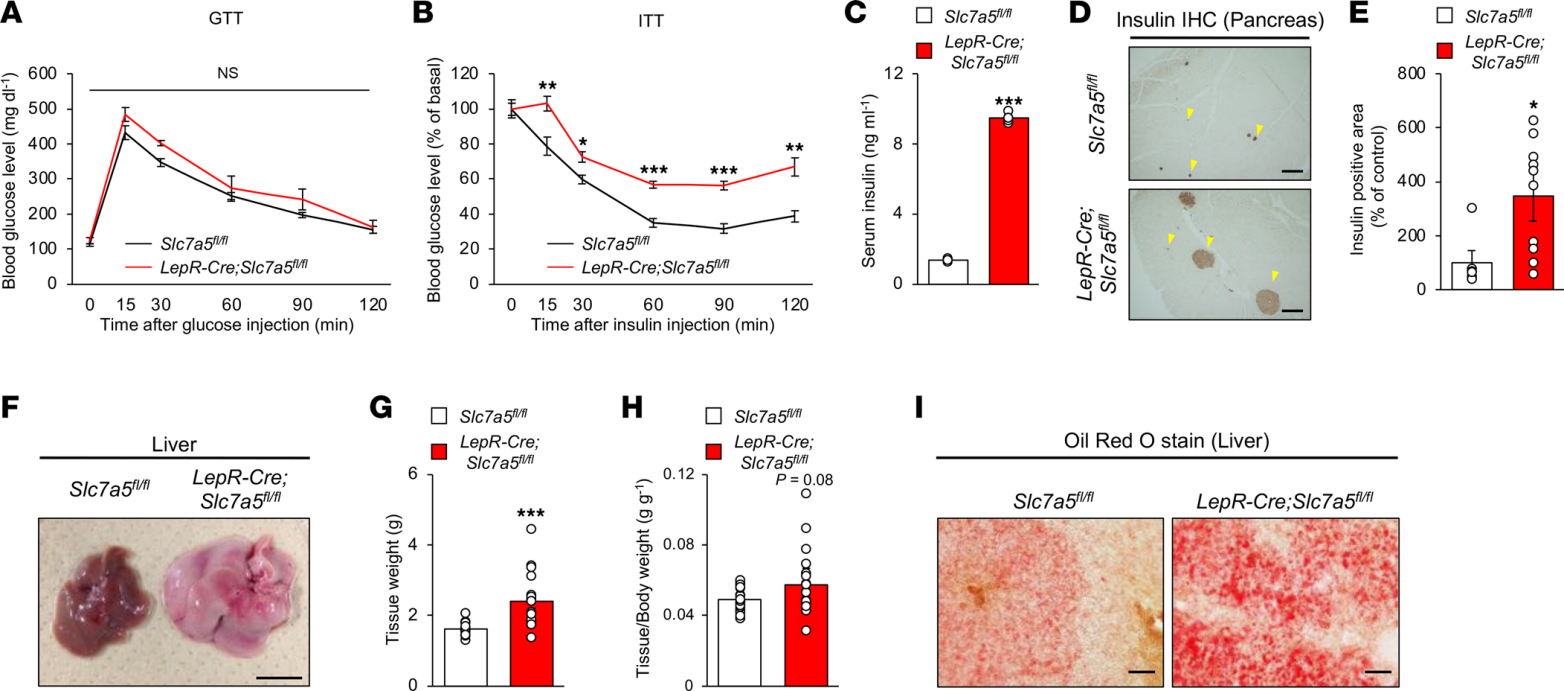
LAT1 in LepR-expressing neurons is involved in insulin sensitivity, energy homeostasis and BAT function. (**A**) Glucose tolerance tests (GTTs) were performed in *LepR-Cre Slc7a5^fl/fl^* mice and control mice after a 6-hour fast at 22–24 weeks of age (*n* = 6 to 8, 2-way ANOVA with Bonferroni post hoc test). (**B**) Insulin tolerance tests (ITTs) were performed in *LepR-Cre Slc7a5^fl/fl^* mice and control mice after a 6-hour fast at 22–24 weeks of age (*n* = 10 or 11, *P < 0.05, **P < 0.01, ***P < 0.001, 2-way ANOVA with Bonferroni post hoc test). (**C**) Serum insulin levels were measured in *LepR-Cre Slc7a5^fl/fl^* mice and control mice after a 6-hour fast at 22–24 weeks of age (*n* = 5, ***P < 0.001, 2-tailed Student’s *t* test). (**D** and **E**) Immunohistochemical stain for insulin was performed on the islets of *LepR-Cre Slc7a5^fl/fl^* mice and control mice at 22–24 weeks of age, followed by quantification of insulin-positive area (*n* = 6 to 10, *P < 0.05, 2-tailed Student’s *t* test). Scale bar, 200 μm. (**F**–**H**) Representative picture of liver (**F**), tissue weights (**G**), and tissue weights normalized to body weight (**H**) are shown for *LepR-Cre Slc7a5^fl/fl^* mice and control mice at 22–24 weeks of age (*n* = 17 or 18, ***P < 0.001, 2-tailed Student’s *t* test). Scale bar, 500 μm. (**I**) Oil Red O stain was performed on the liver of *LepR-Cre Slc7a5^fl/fl^* mice and control mice at 22–24 weeks of age. Scale bar, 100 μm. All the mice used in this study were male.

**Figure 4:**
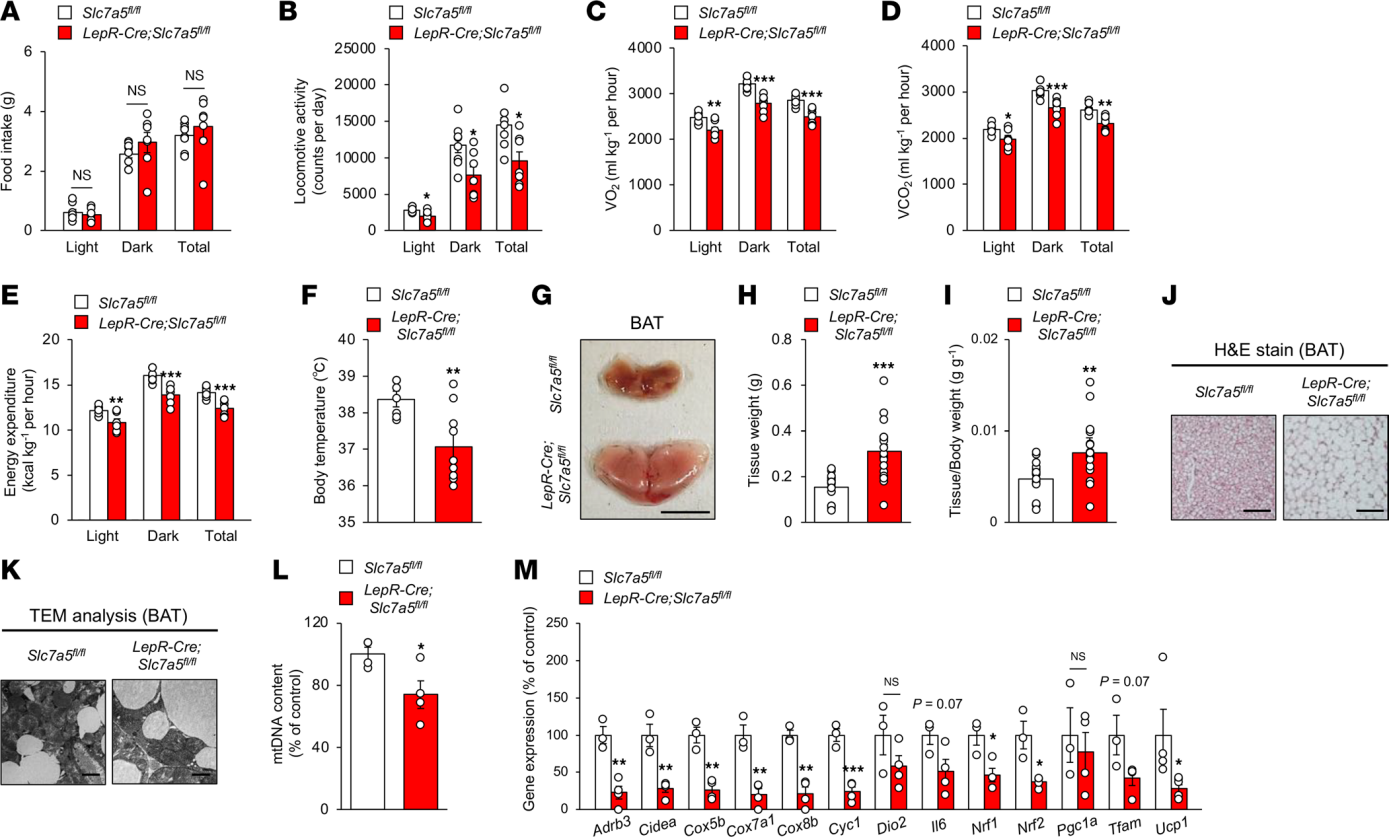
LAT1 in LepR-expressing neurons is involved in energy homeostasis and BAT function. (**A**–**F**) Food intake (**A**), locomotor activity (**B**), O_2_ consumption (**C**), CO_2_ production (**D**), energy expenditure (**E**), and body temperature (**F**) were measured in singly housed *LepR-Cre Slc7a5^fl/fl^* mice and control mice at 22–24 weeks of age (*n* = 7 to 10, *P < 0.05, **P < 0.01, ***P < 0.001, 2-tailed Student’s *t* test). (**G**–**I**) Representative picture of BAT (**G**), tissue weights (**H**), and tissue weights normalized to body weight (**I**) are shown for *LepR-Cre Slc7a5^fl/fl^* mice and control mice at 22–24 weeks of age (*n* = 17 or 18, **P < 0.01, ***P < 0.001, 2-tailed Student’s *t* test). Scale bar, 1 cm. (**J**) H&E stain was performed on the BAT of *LepR-Cre Slc7a5^fl/fl^* mice and control mice at 22–24 weeks of age. Scale bar, 100 μm. (**K**) Transmission electron microscopy analysis was performed on the BAT of *LepR-Cre Slc7a5^fl/fl^* mice and control mice at 22–24 weeks of age. Scale bar, 10 μm. (**L** and **M**) mtDNA content of BAT (**L**) and mRNA expression (**M**) were measured in *LepR-Cre Slc7a5^fl/fl^* mice and control mice at 22–24 weeks of age (*n* = 3 to 4, *P < 0.05, **P < 0.01, ***P < 0.001, 2-tailed Student’s *t* test). All the mice used in this study were male.

We next examined whether LAT1 in LepR-expressing neurons was involved in the regulation of glucose handling and insulin sensitivity. *LepR-Cre Slc7a5^fl/fl^* mice exhibited normal glucose tolerance but impaired insulin sensitivity associated with elevated serum insulin levels and enlarged islets (Figure 3, A–E). *LepR-Cre Slc7a5^fl/fl^* mice also developed severe liver steatosis with marked lipid accumulation (Figure 3, F–I). Food intake was comparable between *LepR-Cre Slc7a5^fl/fl^* and control mice in all phases; however, locomotor activity was significantly decreased in *LepR-Cre Slc7a5^fl/fl^* mice compared with that in control mice in all phases (Figure 4, A and B, and Supplemental Figure 3, A–C). O_2_ consumption, CO_2_ production, and energy expenditure were significantly decreased in *LepR-Cre Slc7a5^fl/fl^* mice compared with those in control mice in all phases (Figure 4, C–E, and Supplemental Figure 3, D–F). Core body temperature was significantly decreased in *LepR-Cre Slc7a5^fl/fl^* mice compared with that in control mice (Figure 4F). Brown adipose tissue (BAT) weight was remarkably higher in *LepR-Cre Slc7a5^fl/fl^* mice and associated with larger lipid infiltration, suggesting whitening of BAT (Figure 4, G–J). In addition, mitochondrial content in BAT was significantly decreased in *LepR-Cre Slc7a5^fl/fl^* mice, and morphological abnormalities of the mitochondria were present (Figure 4, K and L). Furthermore, *LepR-Cre Slc7a5^fl/fl^* mice displayed morphological abnormalities in the mitochondria of the soleus muscle associated with lower mitochondria content (Supplemental Figure 3, G and H). Furthermore, thermogenic and mitochondrial function-related genes, such as *Ucp1*, *Adrb3*, *Tfam*, and *Cox7a*, were downregulated in the BAT of *LepR-Cre Slc7a5^fl/fl^* mice (Figure 4M). Collectively, these results show that LAT1 in LepR-expressing neurons is required for the regulation of insulin sensitivity, energy expenditure, and BAT function but not for glucose handling and energy intake.

### LAT1 in LepR-expressing neurons is implicated in HFD-induced obesity and metabolic dysfunction.

To test whether LAT1 in LepR-expressing neurons contributes to metabolic response to diet-induced obesity, we weekly measured body weight of *LepR-Cre Slc7a5^fl/fl^* mice switched to HFD at 7 weeks of age. *LepR-Cre Slc7a5^fl/fl^* mice gained higher body weight starting from 10 weeks of age, accompanied by enlargement of the liver and BAT (Supplemental Figure 4, A–C). Moreover, *LepR-Cre Slc7a5^fl/fl^* mice displayed significant impairments in glucose tolerance and insulin sensitivity (Supplemental Figure 4, D and E). Together, these results suggest that *Slc7a5* deficiency in LepR-expressing neurons exacerbates HFD-induced obesity and metabolic dysfunction.

### LAT1 in LepR-expressing neurons controls leptin sensitivity and sympathetic tone.

**Figure 5:**
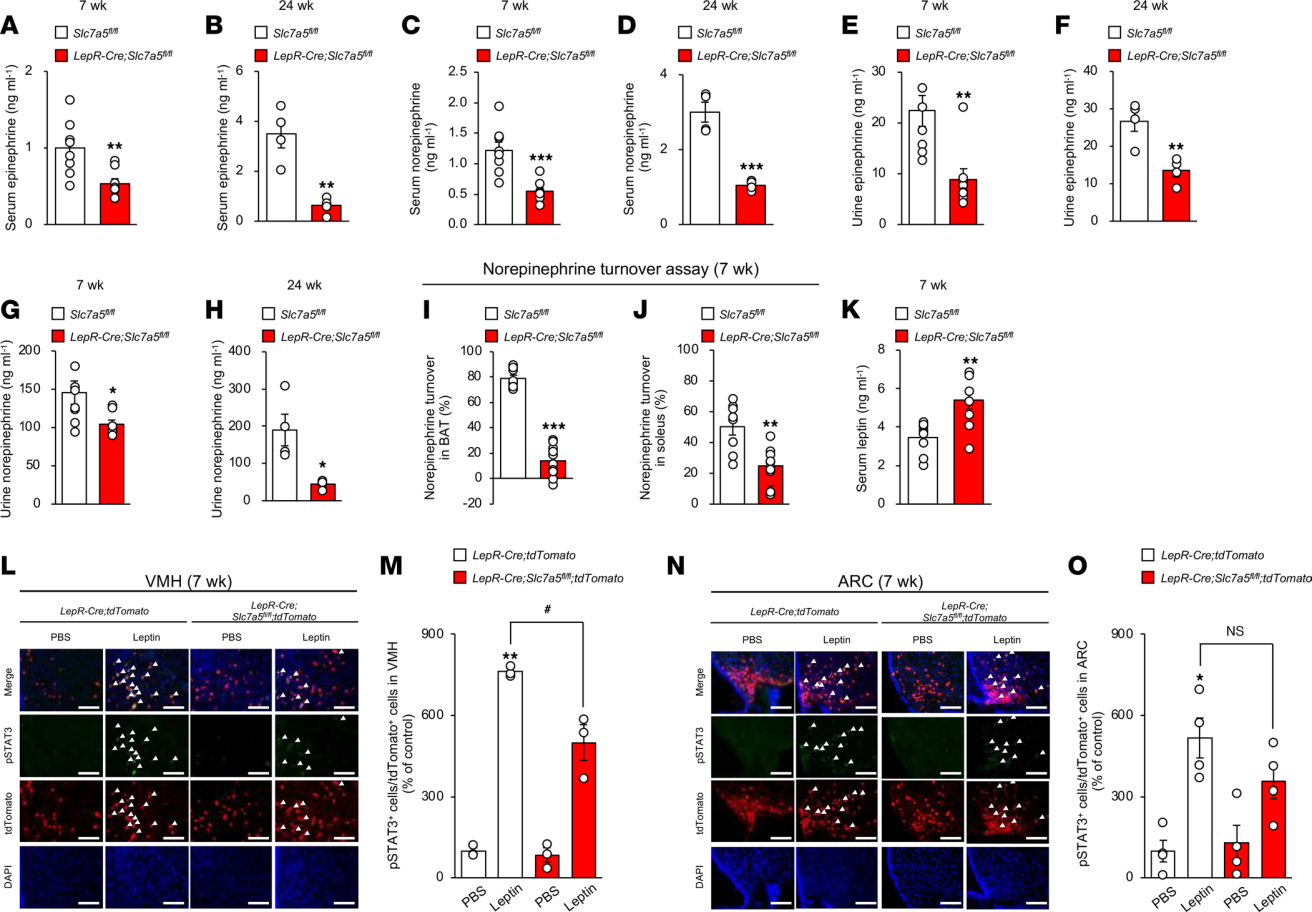
LAT1 in LepR-expressing VMH neurons regulates leptin sensitivity and sympathetic outflow. (**A** and **B**) Serum epinephrine levels were measured in *LepR-Cre Slc7a5^fl/fl^* mice and control mice at 7 (**A**) or 24 (**B**) weeks of age (7 wk; *n* = 8, 24 wk; *n* = 4, **P < 0.01, 2-tailed Student’s *t* test). (**C** and **D**) Serum norepinephrine levels were measured in *LepR-Cre Slc7a5^fl/fl^* mice and control mice at 7 (**C**) or 24 (**D**) weeks of age (7 wk; *n* = 8, 24 wk; *n* = 4, ***P < 0.001, 2-tailed Student’s *t* test). (**E** and **F**) Urine epinephrine levels were measured in *LepR-Cre Slc7a5^fl/fl^* mice and control mice at 7 (**E**) or 24 (**F**) weeks of age (7 wk; *n* = 8, 24 wk; *n* = 4, **P < 0.01, 2-tailed Student’s *t* test). (**G** and **H**) Urine norepinephrine levels were measured in *LepR-Cre Slc7a5^fl/fl^* mice and control mice at 7 (**G**) or 24 (**H**) weeks of age (7 wk; *n* = 8, 24 wk; *n* = 4, *P < 0.05, 2-tailed Student’s *t* test). (**I** and **J**) Norepinephrine turnover levels of (**I**) BAT and (**J**) soleus muscle were measured in *LepR-Cre Slc7a5^fl/fl^* mice and control mice at 24 weeks of age (*n* = 8 to 10, **P < 0.01, ***P < 0.001, 2-tailed Student’s *t* test). (**K**) Serum leptin levels were measured in *LepR-Cre Slc7a5^fl/fl^* mice at 7 weeks of age (*n* = 8, **P < 0.01, 2-tailed Student’s *t* test). (**L**–**O**) Immunohistochemical analysis of phosphorylated STAT3 activation 2 hours after intraperitoneal injection of 5 mg/kg leptin was performed on the VMH (**L** and **M**) and ARC (**N** and **O**) of *LepR-Cre Slc7a5^fl/fl^* mice and control mice at 7 weeks of age (*n* = 3 or 4, *P < 0.05, **P < 0.01, *^#^P* < 0.05, 2-way ANOVA with Bonferroni post hoc test). Scale bar, 100 μm. Arrowheads indicate representative phosphorylated STAT3/tdTomato double-positive neurons. All the mice used in this study were male.

Given that LepR-expressing neurons regulate systemic energy homeostasis via the SNS (36), we next assessed the impact of LAT1 in LepR-expressing neurons on sympathetic nerve activity. Epinephrine and norepinephrine levels were significantly lower in the serum and urine of *LepR-Cre Slc7a5^fl/fl^* mice than in control mice before the onset of obesity (7 weeks of age) and after the diagnosis of obesity (24 weeks of age) (Figure 5, A–H). Furthermore, we performed a norepinephrine turnover assay (37), which reflects the total sympathetic drive onto peripheral target tissues, including BAT and the soleus muscle. *LepR-Cre Slc7a5^fl/fl^* mice displayed a significant decrease in norepinephrine turnover in both BAT and soleus muscle tissue compared with control mice at 7 weeks of age (Figure 5, I and J), thereby indicating that *Slc7a5* deficiency in LepR-expressing neurons led to loss of sympathetic innervation of BAT and muscle before the onset of obesity.

Serum leptin levels were significantly increased in *LepR-Cre Slc7a5^fl/fl^* mice prior to the onset of obesity (7 weeks of age) (Figure 5K) and after the diagnosis of obesity (24 weeks of age) (Figure 2H). This suggests that *Slc7a5* deficiency in LepR-expressing neurons is involved in leptin insensitivity. We therefore hypothesized that LAT1 regulates sympathetic tone by modulating leptin sensitivity (leptin action on LepR-expressing neurons). STAT3 is a well-known direct target of leptin, and its phosphorylation levels reflect leptin sensitivity (3). Systemic leptin administration significantly increased STAT3 phosphorylation in LepR-expressing VMH and ARC neurons. Meanwhile, leptin-induced STAT3 phosphorylation was significantly impaired under *Slc7a5* deficiency in LepR-expressing VMH neurons, but not in ARC neurons, prior to the onset of obesity (Figure 5, L–O).

Taken together, these results indicate that LAT1 in LepR-expressing neurons controls leptin sensitivity and subsequent sympathetic outflow to regulate systemic energy homeostasis. This may represent a cause, rather than a secondary consequence, of obesity.

### LAT1 in LepR-expressing cells regulates bone homeostasis.

**Figure 6:**
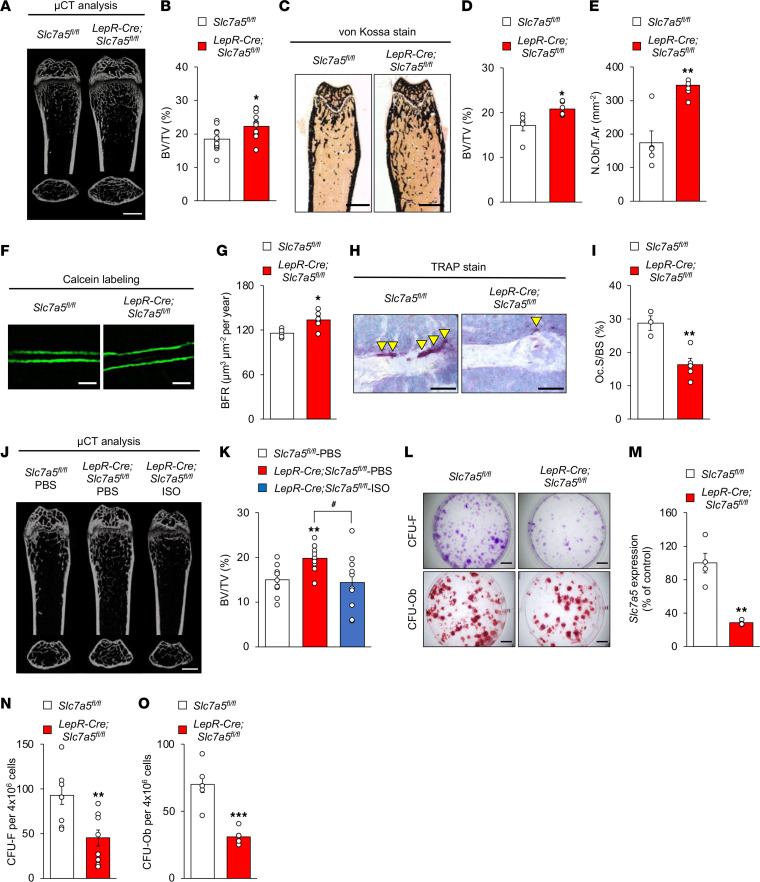
Bone homeostasis is regulated by LAT1 in LepR-expressing cells. (**A**) Micro–computed tomography (μCT) analysis (scale bar, 1 mm); (**B**) bone volume over tissue volume (BV/TV) ratio as determined by μCT (*n* = 12 or 13, *P < 0.05, 2-tailed Student’s *t* test); (**C**) von Kossa stain, scale bar, 1 mm; (**D**) BV/TV ratio as determined by von Kossa stain (*n* = 5 or 6, *P < 0.05, 2-tailed Student’s *t* test); (**E**) number of osteoblasts/tissue area ratio (*n* = 5 or 6, **P < 0.01, 2-tailed Student’s *t* test); (**F**) calcein labeling (scale bar, 50 μm); (**G**) bone formation rate (*n* = 5 or 6, *P < 0.05, 2-tailed Student’s *t* test); (**H**) TRAP stain (scale bar, 50 μm; arrowheads indicate TRAP-positive osteoclasts); and (**I**) osteoclast surface/bone surface ratio of femurs from *LepR-Cre Slc7a5^fl/fl^* mice and control mice at 12–16 weeks of age (*n* = 3 to 5, **P < 0.01, 2-tailed Student’s *t* test). TRAP, tartrate-resistant acid phosphatase. (**J**) μCT analysis, scale bar, 1 mm and (**K**) BV/TV ratio as determined by μCT of femurs from *LepR-Cre Slc7a5^fl/fl^* mice administrated with isoproterenol at 14 weeks of age (*n* = 10, **P < 0.01, ^#^*P* < 0.05, 2-tailed Student’s *t* test with Benjamini-Hochberg correction). (**L**) Representative images of CFU assays stained with crystal violet and alizarin red (scale bar, 500 μm), (**M**) mRNA level of *Slc7a5* in BM-MSCs (*n* = 4, **P < 0.01, 2-tailed Student’s *t* test), and (**N** and **O**) quantification of CFU assays stained with crystal violet (**N**) and alizarin red (**O**) (*n* = 6 to 9, **P < 0.01, ***P < 0.001, 2-tailed Student’s *t* test) in *LepR-Cre Slc7a5^fl/fl^* mice at 6 weeks of age. All the mice used in this study were male.

Sympathetic tone mediates the central regulation of bone mass by leptin (5, 38). Moreover, VMH neurons are known to control bone homeostasis (1, 39). Given that *LepR-Cre Slc7a5^fl/fl^* mice showed impaired sympathetic tone and leptin insensitivity in LepR-expressing VMH neurons prior to the onset of obesity (Figure 5), we reasoned that LAT1 in LepR-expressing neurons may play an important role in regulating bone homeostasis in addition to energy homeostasis. *LepR-Cre Slc7a5^fl/fl^* mice displayed significantly higher bone mass in the femur but not in the vertebrae than control mice (Figure 6, A–D; Supplemental Figure 5, A and B; and Supplemental Table 1). Bone histomorphometric analyses revealed that bone formation indices (osteoblast number and bone formation rate) were significantly higher in *LepR-Cre Slc7a5^fl/fl^* mice than in control mice (Figure 6, E–G, and Supplemental Table 2). Conversely, a bone resorption index (osteoclast surface) was significantly lower in *LepR-Cre Slc7a5^fl/fl^* mice than in control mice (Figure 6, H and I). We next assessed whether the impairment of sympathetic outflow is involved with the abnormal bone homeostasis of *LepR-Cre Slc7a5^fl/fl^* mice. Systemic administration of isoproterenol, a β-adrenoreceptor agonist, corrected the higher bone mass phenotype of *LepR-Cre Slc7a5^fl/fl^* mice (Figure 6, J and K, and Supplemental Table 3). *LepR-Cre* mice did not display any abnormalities in bone mass compared to control mice (Supplemental Figure 5, C and D, and Supplemental Table 4).

LepR is also a marker that is highly enriched in bone marrow mesenchymal stem cells (BM-MSCs), and LepR-expressing BM-MSCs represent a major source of bone in adult bone marrow (40). The numbers of both colony-forming unit fibroblasts (CFU-F) and CFU osteoblasts (CFU-Ob) were significantly reduced under *Slc7a5* deficiency in BM-MSC culture in vitro, indicating decreased stemness and osteogenic potential of MSCs, which contradicted the high bone mass phenotype of *LepR-Cre Slc7a5^fl/fl^* mice (Figure 6, L–O).

These results raise a possibility that LAT1 in LepR-expressing neurons centrally regulates bone homeostasis in *LepR-Cre Slc7a5^fl/fl^* mice.

### LepR-expressing neurons are critical for LAT1-dependent regulation of energy and bone homeostasis.

**Figure 7:**
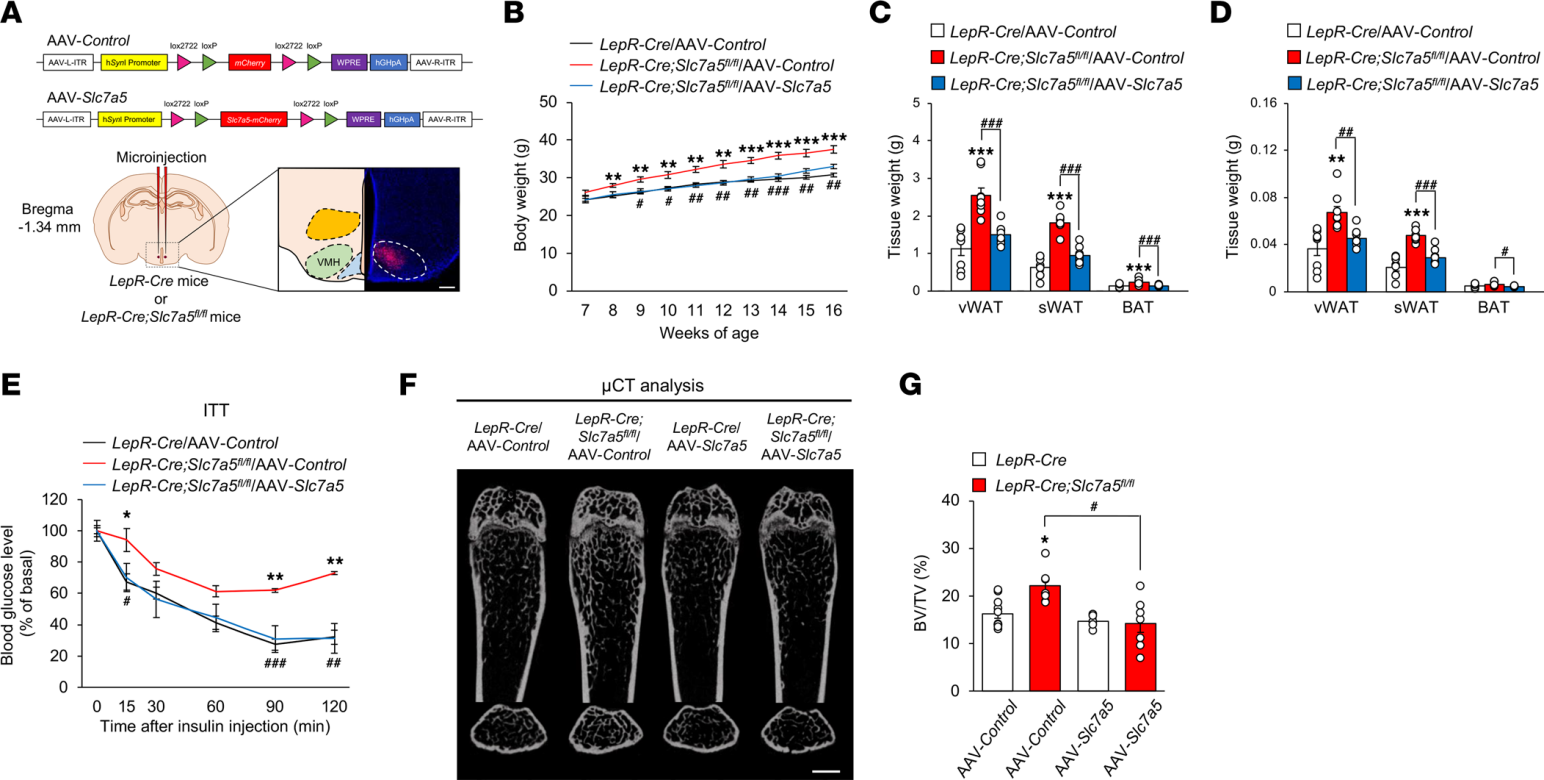
LepR-expressing VMH neurons contribute to LAT1-dependent regulation of systemic energy and bone homeostasis. (**A**) Schematic diagram of the bilateral viral microinjection into the VMH and representative validation image of mCherry expression in the VMH. Scale bar, 500 μm. (**B**) Weekly body weight after injection of AAV-*Control* or AAV-*Slc7a5* into the VMH is shown for *LepR-Cre Slc7a5^fl/fl^* mice and control mice (*n* = 8 to 12, **P < 0.01, ***P < 0.001: versus *LepR-Cre*/AAV-*Control*, ^#^*P* < 0.05, ^##^*P* < 0.01, ^###^*P* < 0.001: versus *LepR-Cre Slc7a5^fl/fl^*/AAV-*Control*, 2-way ANOVA with Bonferroni post hoc test). (**C** and **D**) Adipose tissue weights (**C**) and adipose tissue weights normalized to body weight (**D**) are shown for *LepR-Cre Slc7a5^fl/fl^* mice and control mice injected with AAV-*Control* or AAV-*Slc7a5* into the VMH at 16 weeks of age (*n* = 8, **P < 0.01, ***P < 0.001, ^#^*P* < 0.05, ^##^*P* < 0.01, ^###^*P* < 0.001, 2-tailed Student’s *t* test with Bonferroni correction). (**E**) ITTs were performed in *LepR-Cre Slc7a5^fl/fl^* mice and control mice injected with AAV-*Control* or AAV-*Slc7a5* into the VMH after a 6-hour fast at 16 weeks of age (*n* = 3 or 4, *P < 0.05, **P < 0.01: versus *LepR-Cre*/AAV-*Control*, ^#^*P* < 0.05, ^##^*P* < 0.01, ^###^*P* < 0.001: versus *LepR-Cre Slc7a5^fl/fl^*/AAV-*Control*, 2-way ANOVA with Bonferroni post hoc test). (**F**) μCT analysis (scale bar, 1 mm) and (**G**) BV/TV ratio as determined by μCT of femurs from *LepR-Cre Slc7a5^fl/fl^* mice and control mice injected with AAV-*Control* or AAV-*Slc7a5* into the VMH at 12–16 weeks of age (*n* = 6 to 11, *P < 0.05, ^#^*P* < 0.05, 2-way ANOVA with Bonferroni post hoc test). All the mice used in this study were male.

The VMH is a central site for homeostatic regulation of various phenotypes, including those of energy and bone (1, 39, 41–43). In addition, the fact that leptin insensitivity was observed in VMH neurons of *LepR-Cre Slc7a5^fl/fl^* mice before the onset of obesity (Figure 5) alongside impairment in BCAA uptake in the VMH (Figure 2) led us to examine whether LAT1 in LepR-expressing VMH neurons was indeed important for the control of energy and bone homeostasis. To this end, we selectively reexpressed *Slc7a5* in LepR-expressing neurons by stereotaxically and bilaterally microinjecting a Cre-inducible adeno-associated virus (AAV), *AAV-hSynI-DIO-Slc7a5-mCherry* (AAV-*Slc7a5*), into the VMH of *LepR-Cre Slc7a5^fl/fl^* mice before the onset of obesity (Figure 7A). Viral introduction of *Slc7a5* in LepR-expressing VMH neurons almost completely reduced the body weight gain, fat pad accumulation, and insulin insensitivity observed in *LepR-Cre Slc7a5^fl/fl^* mice 9 weeks after AAV injection (Figure 7, B–E). In contrast, *Slc7a5* reexpression in the ARC neurons of *LepR-Cre Slc7a5^fl/fl^* mice did not counteract the abnormal energy homeostasis (Supplemental Figure 6, A–F). Moreover, viral introduction of *Slc7a5* in the VMH neurons of *LepR-Cre Slc7a5^fl/fl^* mice corrected the high bone mass phenotype, suggesting that LAT1 in LepR-expressing neurons centrally regulates bone mass accrual rather than cell-intrinsic action in bone tissue (Figure 7, F and G, and Supplemental Table 5).

These results demonstrate that LAT1 in LepR-expressing VMH neurons contributes to energy and bone homeostasis.

### mTORC1 is a major regulator of energy and bone homeostasis downstream of LAT1.

**Figure 8:**
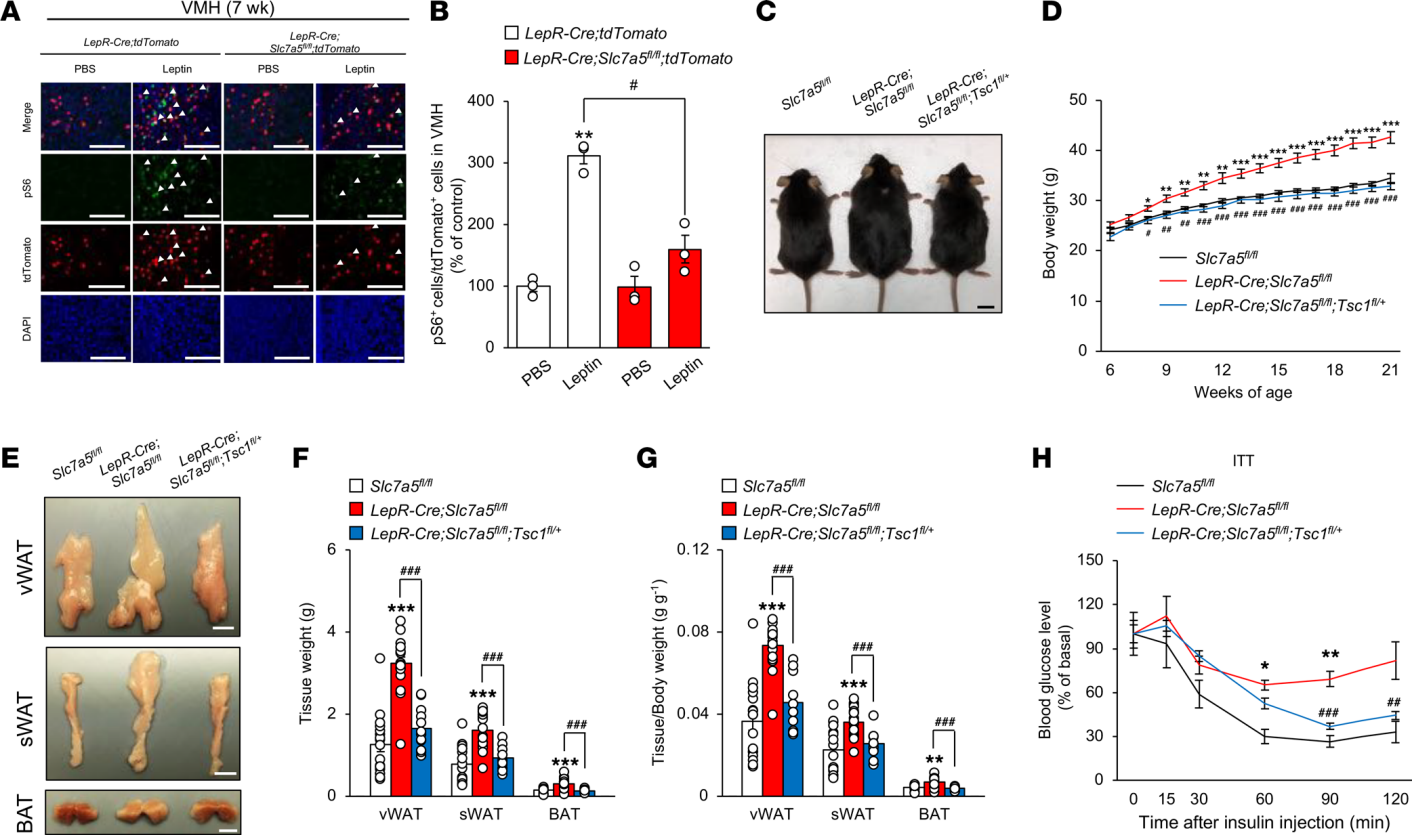
mTORC1 is a critical regulator of systemic energy homeostasis downstream of LAT1. (**A** and **B**) Immunohistochemical analysis of pS6 activation 2 hours after intraperitoneal injection of 5 mg/kg leptin was performed on the VMH of *LepR-Cre Slc7a5^fl/fl^* mice and control mice at 7 weeks of age (*n* = 3, **P < 0.01, ^#^*P* < 0.05, 2-way ANOVA with Bonferroni post hoc test). Scale bar, 100 μm. Arrowheads indicate pS6/tdTomato double-positive neurons. (**C**) Gross appearance at 24 weeks of age is shown for *LepR-Cre Slc7a5^fl/fl^* mice, *LepR-Cre Slc7a5^fl/fl^ Tsc1^fl/+^* mice, and control mice fed an NC. Scale bar, 1 cm. (**D**) Weekly body weight is shown for *LepR-Cre Slc7a5^fl/fl^* mice, *LepR-Cre Slc7a5^fl/fl^ Tsc1^fl/+^* mice, and control mice fed NC (*n* = 9 to 11, *P < 0.05, **P < 0.01, ***P < 0.001: versus *Slc7a5^fl/fl^*, ^#^*P* < 0.05, ^##^*P* < 0.01, ^###^*P* < 0.001: versus *LepR-Cre Slc7a5^fl/fl^*, 2-way ANOVA with Bonferroni post hoc test). (**E**–**G**) Representative pictures of vWAT, sWAT, and BAT (**E**); adipose tissue weights (**F**); and adipose tissue weights normalized to body weight (**G**) are shown for *LepR-Cre Slc7a5^fl/fl^* mice and control mice at 22–24 weeks of age (*n* = 12 to 18, **P < 0.01, ***P < 0.001, ^###^*P* < 0.001, 1-way ANOVA with Bonferroni post hoc test). Scale bar, 1 cm. (**H**) ITTs were performed in *LepR-Cre Slc7a5^fl/fl^* mice, *LepR-Cre Slc7a5^fl/fl^ Tsc1^fl/+^* mice, and control mice after a 6-hour fast at 22–24 weeks of age (*n* = 3 to 9, *P < 0.05, **P < 0.01: versus *Slc7a5^fl/fl^*, ^##^*P* < 0.01, ^###^*P* < 0.001: versus *LepR-Cre Slc7a5^fl/fl^*, 2-way ANOVA with Bonferroni post hoc test). All the mice used in this study were male.

LAT1 facilitates cellular uptake of BCAAs, including leucine, leading to stimulation of mTORC1 signaling (20, 21). mTORC1 signaling, associated with leptin signaling in the hypothalamus, is implicated in the regulation of energy homeostasis, alongside contributing to leptin resistance (32, 44). These previous findings led us to assess whether mTORC1 signaling, which can be affected by leptin, participates in LAT1-dependent regulation of energy and bone homeostasis. Systemic leptin administration significantly increased phosphorylation of ribosomal protein S6, an indicator of mTORC1 activity (45), in LepR-expressing VMH neurons. In addition, leptin-induced mTORC1 activation was significantly suppressed under *Slc7a5* deficiency prior to the onset of obesity (Figure 8, A and B).

**Figure 9:**
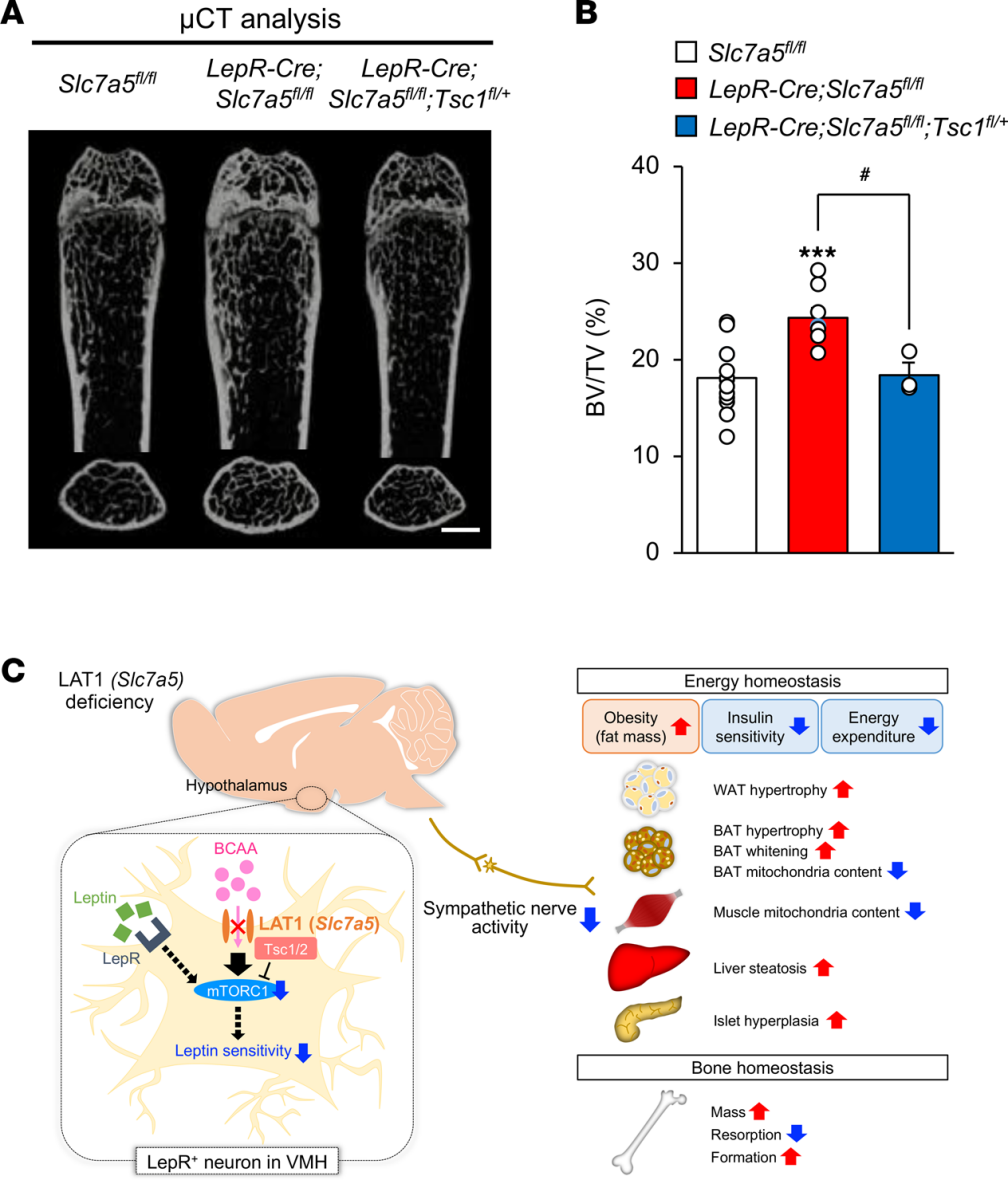
mTORC1 is a critical regulator of bone homeostasis downstream of LAT1. (**A**) μCT analysis, scale bar, 1 mm; and (**B**) BV/TV ratio as determined by μCT of femurs from *LepR-Cre Slc7a5^fl/fl^* mice, *LepR-Cre Slc7a5^fl/fl^ Tsc1^fl/+^* mice, and control mice at 12–16 weeks of age (*n* = 3 to 14, ***P < 0.001, ^#^*P* < 0.05, 1-way ANOVA with Bonferroni post hoc test). (**C**) Working model of the findings of this study. All the mice used in this study were male.

Tuberous sclerosis complex 1 (Tsc1), also known as hamartin, is a critical negative regulator of mTORC1 (45). To assess whether mTORC1 signaling is directly involved in the abnormal regulation of energy and bone homeostasis observed in *LepR-Cre Slc7a5^fl/fl^* mice, we generated *LepR-Cre Slc7a5^fl/fl^* mice lacking 1 copy of the floxed *Tsc1* allele (*LepR-Cre Slc7a5^fl/fl^ Tsc1^fl/+^*). The obesity, fat accumulation, and insulin insensitivity in *LepR-Cre Slc7a5^fl/fl^* mice were ameliorated in *LepR-Cre Slc7a5^fl/fl^ Tsc1^fl/+^* mice (Figure 8, C–H). Moreover, the high bone mass phenotype of *LepR-Cre Slc7a5^fl/fl^* mice was almost completely corrected in *LepR-Cre Slc7a5^fl/fl^ Tsc1^fl/+^* mice (Figure 9, A and B, and Supplemental Table 6). *LepR-Cre Tsc1^fl/+^* mice did not display any abnormalities in body weight, glucose tolerance, insulin sensitivity, adipose tissue weights, and bone mass compared to control mice (Supplemental Figure 7, A–G, and Supplemental Table 7).

Taken together, these results indicate that LAT1 in LepR-expressing VMH neurons contributes to energy and bone homeostasis through mTORC1 signaling.

## Discussion

This study revealed that genetic inactivation of LAT1 in LepR-expressing neurons led to metabolic and skeletal abnormalities. These abnormalities may have been consequences of decreased amino acid uptake and leptin insensitivity in neurons and subsequent inactivation of sympathetic outflow to target organs. To the best of our knowledge, this is the first study to indicate that LAT1 in LepR-expressing neurons is the primary and central amino acid sensor involved in the co-regulation of energy and bone homeostasis (Figure 9C).

There are 2 major intracellular amino acid–sensing mechanisms: 1) the general amino acid control (GAAC) pathway mediated by general control nonderepressible 2 (GCN2) and activating transcription factor 4 and 2) the mTORC1 pathway (46–48). It is controversial whether GCN2 expressed in the brain can sense amino acids in the diet (49–51). mTORC1 acts as a sensor of energy status in the hypothalamus in a neuron type– and context-dependent manner (4). The activity of mTORC1 is suppressed in the VMH under a fasting state or in leptin-deficient *ob*/*ob* mice (52, 53), whereas refeeding markedly decreases mTORC1 activity in the VMH in both lean and obese rats (54). Moreover, hypothalamic PI3K/mTORC1 signaling is critical for mediating the SNS effects of leptin (55). Given that genetic activation of mTORC1 almost completely corrected both the metabolic abnormalities and the high bone mass in *Slc7a5*-deficient mice, our findings suggest that mTORC1 signaling rather than the GAAC pathway is the pivotal effector downstream of LAT1 in LepR-expressing VMH neurons.

VMH neurons are central to the balance between energy and bone metabolism. Indeed, electric and chemical lesioning and cell-specific gene deletion in VMH neurons can incur an imbalance in energy and bone homeostasis (1, 38, 39, 56). In this study, *Slc7a5* deficiency led to leptin insensitivity in LepR-expressing neurons and decreased sympathetic outflow prior to the onset of obesity. Notably, preferential reexpression of *Slc7a5* in LepR-expressing VMH neurons almost completely corrected the high bone mass phenotype as well as metabolic abnormalities in *Slc7a5*-deficient mice, indicating that LAT1 in LepR-expressing VMH neurons contributes to the control of energy and bone homeostasis through modulation of sympathetic outflow to target organs. Given that LepR is expressed in a variety of regions within the brain, including the midbrain, brainstem, and hypothalamus (57), we could not exclude the possibility that LAT1 in LepR-expressing neurons in other brain areas is involved in energy and bone homeostasis. Future research will be required to characterize the role of LAT1 in different hypothalamic regions using *Sf1-Cre*, *Agrp-Cre*, and *Pomc-Cre* mice (58–60).

Further studies are necessary to identify the specific neural circuit whereby energy and bone metabolism is regulated by the LAT1/mTORC1 axis in LepR-expressing neurons. VMH neurons send direct neuronal projections to the subregion of the nucleus tractus solitarius, which is known to modulate sympathetic activity (39). The paraventricular nucleus of the hypothalamus is a known sympathetic premotor region in the CNS that regulates energy homeostasis (61, 62). Moreover, the rostral raphe pallidus in the rostral ventromedial medulla projects to BAT sympathetic preganglionic neurons in the spinal intermediolateral nucleus (63). Accordingly, these regions could represent intermediate neuronal populations through which the LAT1/mTORC1 axis in VMH neurons regulates energy and bone homeostasis through sympathetic tone.

It should be emphasized that the LAT1-dependent amino acid uptake system is operational in the hypothalamus. We were unable to precisely identify the neuron types in which LAT1 was operational in the hypothalamus owing to limitations of radioisotope-labeled amino acid uptake experiments. However, the downregulation of LAT1-dependent amino acid uptake in the hypothalamus of the obese/diabetic mouse model points to a possible inverse association between LAT1 function in the hypothalamus and the impairment of energy homeostasis, although the mechanisms governing LAT1 function in the hypothalamus remain unclear.

In conclusion, the current findings highlight a critical mechanism underlying energy and bone homeostasis via the amino acid sensor LAT1 in LepR-expressing neurons. Although the effect of BCAA-rich status on energy and bone homeostasis in *Slc7a5*-deficient mice should be examined to further define the physiological role of LAT1 in LepR-expressing neurons, this represents a potentially novel molecular connection among a nutritional signal in the CNS, energy homeostasis, and skeletal integrity. LAT1 has been proposed as a promising target for diagnostic cancer imaging and anticancer therapeutics (64, 65). We suggest that manipulation of the hypothalamic LAT1/mTORC1 axis represents a plausible diagnostic and therapeutic strategy for coordinating the sophisticated energy balance and skeletal integrity required for protection against osteoporosis and obesity in humans.

## Methods

### Mice.

*Tsc1^fl/fl^* mice (66) (005680), *Rosa26-tdTomato* mice (67) (007908), and *LepR-Cre* mice (68) (008320) were obtained from Thr Jackson Laboratory. *db/db* mice were obtained from Japan SLC. *Slc7a5^fl/fl^* mice, *Tsc1^fl/fl^* mice, and *Rosa26-tdTomato* mice were crossed with *LepR-Cre* mice. These mutant mice were backcrossed more than 5 generations with C57BL/6J. Mice were bred under standard animal housing conditions at 23 ± 1°C with humidity of 55% and a 12-hour light/12-hour dark cycle, with free access to either normal chow or HFD (Research Diets, D12492; 60% from fat, 5.24 kcal/g) and water. To measure the effects of isoproterenol (FUJIFILM Wako Pure Chemical I874200), mice at 12 weeks of age were used and were administrated with isoproterenol (15 mg/kg of body weight, daily i.p. injection) for 2 weeks. All the studies described here used male mice. Genotyping was performed by PCR using tail genomic DNA with specific primers (Supplemental Table 8). The study protocol meets the guidelines of the Japanese Pharmacological Society and was approved by the Committee for the Ethical Use of Experimental Animals at Gifu Pharmaceutical University, Gifu University, and Kanazawa University. The number of animals used per experiment is stated in the figure legends.

### Validation of deletion efficiency and specificity.

Brain samples were coronally sliced to a thickness of 1.0 mm (bregma, –0.5 to –2.5 mm) according to *Paxinos and Franklin’s the Mouse Brain in Stereotaxic Coordinates* (69) with Brain Matrix (ASI Instruments, RBM-2000C), and each region of the hypothalamus (ARC and VMH) was microdissected using a razor blade (Electron Microscopy Sciences). Genomic DNA and RNA were extracted from each microdissected region of hypothalamus, adipose tissue (vWAT, sWAT, and BAT), muscle, and liver, followed by PCR with specific primers (Supplemental Table 9).

### Metabolic study and physiological measurements.

GTTs were conducted as follows. Glucose (2 g/kg) was injected intraperitoneally into mice after a 6-hour fast, and blood glucose levels were monitored using blood glucose strips and an Accu-Check glucometer (Roche) at the indicated times. For ITTs, insulin (1.5 U/kg) was injected intraperitoneally into mice after a 6-hour fast, and blood glucose levels were monitored as described previously (70).

Metabolic rate was measured by indirect calorimetry in mice using Oxymax/CLAMS (Columbus Instruments). Mice were individually housed in chambers maintained at 24 ± 1°C and given free access to food (normal chow) and water. The colonic temperature was measured with a rectal thermometer (Natsume Seisakusho, KN-91). During the study, samples were taken at 13-minute intervals to measure O_2_ consumption and CO_2_ production and to calculate energy expenditure. Food intake and locomotive activity were automatically measured every 20 minutes by the cFDM-300AS system (MELQUEST Ltd.).

### Bone histomorphometric analysis and x-ray μCT analysis.

Bone histomorphometric analyses were performed on undecalcified long bones and vertebrae in accordance with previously described methods (71). Briefly, the bone was fixed with 4% paraformaldehyde, followed by dehydration in an ethanol series, and subsequently embedded in methyl methacrylate resin. The BV/TV ratio was measured by von Kossa staining. Osteoblast and osteoclast parameters were analyzed by toluidine blue staining (FUJIFILM Wako Pure Chemical 1B481) and TRAP staining (Cosmo Bio PMC-AK04F-COS), respectively. Analyses were performed using the OsteoMeasure analysis system (OsteoMetrics), according to standard protocols. Trabecular architecture was assessed in long bones using a μCT system (Comscan) at 90 kV and 45 μA, and the BV/TV ratio was measured using TRI/3D-BON software (RATOC) (25). Calcein was injected twice into mice with an interval of 3 days, and then, mice were sacrificed 2 days after the last injection.

### Real-time quantitative PCR.

Total RNA was extracted from the tissues using an RNA extraction kit (Nippon Genetics FG-80250), followed by cDNA synthesis using reverse transcriptase (Thermo Fisher Scientific 28025013) and oligo-dT primers. The cDNA samples were then used as templates for real-time PCR, which was performed on the MX3000P instrument (Agilent Technologies), by using specific primers for each gene (Supplemental Table 10). Dissociation curves for each PCR were used to confirm the specificity of the amplification. The levels of the gene transcripts examined were normalized using *Gapdh* as an internal control for each sample (72). For mtDNA quantification, total DNA containing nuclear DNA and mtDNA was extracted from tissues, followed by purification by phenol/chloroform extraction and EtOH precipitation. Real-time PCR was performed as described above to quantify mtDNA using specific primers for mtDNA and nuclear DNA (Supplemental Table 11). The copy number of mtDNA was normalized with nuclear DNA.

### [^125^I]IMT uptake assay.

[^125^I]IMT, which we synthesized, uptake assay was performed as previously described (25). High-performance liquid chromatography (HPLC) was performed with a COSMOSIL 5C18-AR-II column (4.6 inner diameter [ID] mm × 150 mm; Nacalai Tesque) at a flow rate of 1 mL/min with a gradient mobile phase of 20% methanol in water with 0.1% trifluoroacetic acid (TFA) to 40% methanol in water with 0.1% TFA for 20 minutes. The column temperature was maintained at 40°C. Microdissected ARC/VMH-rich region of hypothalamus was washed twice with HBSS and subsequently incubated in HBSS at 37°C for 10 minutes in a 5% CO_2_ incubator. Samples were then incubated with [^125^I]IMT at 4 or 37°C for 30 minutes. Samples were treated with JPH203 (MilliporeSigma SML1892) at 30 μM. The reaction was terminated by the aspiration of buffer, followed by superficial rinsing with ice-cold HBSS containing 1 mM unlabeled l-α-methyltyrosine (MilliporeSigma 286680) at 4°C 3 times to remove extracellular [^125^I]IMT. Samples were lysed using 0.1 M NaOH. The radioactivity was measured by a γ-counter (Hitachi Ltd., ARC-7010). Protein concentration was determined with a Protein Assay Bicinchoninate Kit (Nacalai Tesque).

### Cell culture.

HEK293T cells were purchased from RIKEN Cell Bank. These cells were cultured at 37°C in a 5% CO_2_ incubator and maintained in DMEM supplemented with 10% fetal bovine serum (FBS) and 1% penicillin/streptomycin.

Primary BM-MSCs were isolated as follows. Briefly, the femur and tibia were isolated and soft tissue was removed. The epiphyses were removed and bone marrow cells were collected by centrifugation at 300*g* for 5 minutes at room temperature. Red blood cells were lysed with ammonium chloride. Cells were cultured at a density of 4 × 10^6^ cells/well in 6-well plates with α-MEM supplemented with 20% FBS and 1% penicillin/streptomycin at 37°C in a 5% CO_2_ incubator. For CFU-F assays, after 14 days in culture, CFU-F colonies were stained by crystal violet solution (FUJIFILM Wako Pure Chemical 038-04862) and counted. For CFU-Ob assays, after 5 days in culture, osteoblast differentiation was initiated by replacing the medium with α-MEM supplemented with 10% FBS, 50 μg/mL ascorbic acid, 10 mM β-glycerophosphate, and 1% penicillin/streptomycin for 14 days. CFU-Ob colonies were assessed by alizarin red staining (FUJIFILM Wako Pure Chemical 011-01192). Clusters of more than 50 cells were considered colonies.

### Amino acid analysis.

The concentrations of amino acids in the VMH- and ARC-rich regions and plasma were measured by liquid chromatography-tandem mass spectrometry (LC-MS/MS) after derivatization with 3-aminopyridyl-*N*-hydroxysuccinimidyl carbamate (APDS) in accordance with a previous method (73), with minor modifications. For the measurement of amino acids in the VMH- and ARC-rich regions of hypothalamus, tissue samples were quickly homogenized with 300 μL of ice-cold acetonitrile, containing gabapentin, used as internal standard. For the measurement of amino acids in plasma samples, 5 μL of plasma samples were mixed with 195 μL of ice-cold methanol, containing gabapentin. The mixture was centrifuged at 25,000*g* for 10 minutes at 4°C to precipitate proteins. Forty μL of the supernatant was mixed with 40 μL of 90 mM sodium borate buffer (pH 8.8) and 10 μL of APDS solution (5 mg/mL in dried acetonitrile). The mixture was incubated at 55°C for 1 hour, followed by addition of 10 μL of 60 mM aspartic acid solution and subsequent incubation for further 60 minutes. Then, the mixture was added with 90 μL of water containing 0.1% formic acid to terminate the reaction.

The APDS-reacted samples were subjected to LC-MS/MS, consisting of a Nexera HPLC system (Shimadzu) coupled to a triple-quadrupole mass spectrometer (Shimadzu, LCMS-8040), using an L-column2 ODS (3 μm, 2.0 mm ID × 150 mm, Chemicals Evaluation and Research Institute). The column temperature was maintained at 50°C, and the flow rate was 0.4 mL/min. The mobile phases were (A) 10 mM ammonium formate in water and (B) 5 mM ammonium formate in methanol. The gradient elution was performed as follows: 0–1.0 minutes, 99% A/1% B; 1.0–5.0 minutes, 99% A/1% B to 80% A/20% B; 5.0–9.0 minutes, 80% A/20% B to 71% A/29% B; 9.0–16.0 minutes, 71% A/29% B to 69% A/31% B; 16.0–20.0 minutes, 69% A/31% B to 5% A/95% B; 20.0–21.0 minutes, 5% A/95% B; 21.0–21.2 minutes, 5% A/95% B to 99% A/1% B; 21.2–25.0 minutes, 99% A/1% B. The autosampler temperature was maintained at 4°C, and the injection volume was 3 μL. Multiple reaction monitoring in positive ion mode was set at 253.0 > 121.0 for Asp-APDS, 226.0 > 121.0 for Ser-APDS, 196.0 > 121.0 for Gly-APDS, 267.0 > 121.0 for Gln-APDS, 276.0 > 156.1 for His-APDS, 210.0 > 121.0 for Ala-APDS, 240.0 > 121.0 for Thr-APDS, 236.0 > 121.0 for Pro-APDS, 302.0 > 121.0 for Tyr-APDS, 238.0 > 121.0 for Val-APDS, 270.0 > 121.0 for Met-APDS, 325.1 > 121.0 for Trp-APDS, 252.1 > 121.0 for Leu-APDS and Ile-APDS, 286.1 > 121.0 for Phe-APDS, 292.1 > 121.0 for gabapentin-APDS. Although the molecular weight of leucine and isoleucine is the same, these 2 amino acids were separated by the column. Data analyses were carried out using LabSolutions Insight (Shimadzu, ver. 3.5) and quantified using sample peak height.

### Hormone measurements.

The levels of leptin, insulin, norepinephrine, and epinephrine were measured using a mouse leptin ELISA kit (R&D Systems, 498-OB), mouse insulin ELISA kit (Abcam ab277390), and Mouse Epinephrine/Norepinephrine ELISA Kit (Abnova, KA1877), respectively.

### Norepinephrine turnover assay.

Norepinephrine turnover was assessed by measuring the decrease in the organ norepinephrine concentration after α-methyltyrosine injection, as described previously (37). Mice were sacrificed by cervical dislocation 0 and 3 hours after α-methyltyrosine injection (300 mg/kg, i.p.), and BAT and soleus muscle were rapidly removed. Organ samples were homogenized in ice-cold 0.2 M perchloric acid containing 0.1 mM EDTA. After the removal of proteins by centrifugation, samples were placed at –80°C until analysis. The samples were analyzed by BIO MEDICAL LABORATORIES profiling service using an HPLC system.

### Transmission electron microscopy.

Tissues were fixed in a solution of 4% paraformaldehyde and 0.5% glutaraldehyde/0.1 M cacodylate. Osmium tetroxide fixation was performed to stabilize lipids following paraformaldehyde/glutaraldehyde fixation. After fixation, samples were dehydrated in increasing concentrations of ethanol. Then dehydrated samples were embedded in Quetol651 resin (Nisshin EM), followed by polymerization at 60°C. The 80 nm ultrathin sections were prepared using a diamond knife (ULTRA, 2.5 mm width, angle 45°, Nisshin EM) equipped with ultracut UCT ultramicrotome (Leica Microsystems) and then mounted on nickel grids (EM fine grid F100, 219 μm 263-N, Nisshin EM). The sections were stained with uranyl acetate, followed by lead citrate to enhance contrast. These processes were done by dipping the grids in droplets of stain, followed by water rinses. After washing with pure water, the grids were imaged with a transmission electron microscope (1200EX; JEOL Ltd. or H7650; Hitachi High-Technologies Corp.) by using the transmission electron image mode and an accelerating voltage of 80 kV.

### Generation of AAV vector and microinjection of AAV.

*pAAV*-*hSynI*-*DIO*-*HA*-*Slc7a5*-*mCherry* vector was generated by replacing *rM3D(Gs)* cDNA in *pAAV-hSyn-DIO-rM3D(Gs)-mCherry* (Addgene 50458, a gift from Bryan Roth, University of North Carolina at Chapel Hill, Chapel Hill, North Carolina, USA) with a mouse *Slc7a5* cDNA from *pCMV6*-*Slc7a5* (Origene, MC217270) vector. *pAAV*-*hSynI*-*DIO*-*HA*-*mCherry* vector was generated by deleting *rM3D(Gs)* cDNA from *pAAV-hSyn-DIO-rM3D(Gs)-mCherry*. Recombinant AAV vectors (AAV-rh10) expressing *mCherry* (AAV-*Control*) or *Slc7a5* (AAV-*Slc7a5*) in a Cre-dependent manner were produced using a triple-transfection, helper-free method and purified as described previously (74). The titers of recombinant AAV vectors were determined by quantitative PCR: AAV-*Control*, 1 × 10^12^; AAV-*Slc7a5*, 1 × 10^12^ genome copies/mL. For microinjection, a small incision was made to expose the skull and drilled at the injection site under aseptic environments using 3 types of mixed anesthetic agents at 7 weeks of age as previously described. AAVs were injected at a rate of 100 nL/min for 10 minutes into the VMH (bregma: *x*: ±0.3 mm, *y*: –1.46 mm, *z*: 5.5 mm) using a micromanipulator (MUROMACHI KIKAI, IMPACT-1000), and the injector remained in place for an additional 5 minutes before removal.

### Bioinformatics.

For mouse studies, we used the RNA-Seq data of VMH and ARC derived from normal chow– or HFD-fed wild-type mice deposited in the National Center for Biotechnology Information (NCBI) Gene Expression Omnibus (GEO) database (GSE148641) (75). Data acquisition and quality check were performed using SRA Toolkit (v2.10.4) and FastQC (v0.11.8), respectively. Then, we aligned sequence reads with STAR (v2.7.3a) and calculated read counts and transcripts per million (TPM) by RSEM (v1.3.3) against the mouse reference sequence (GRCm38). Calculating counts per million and differential expression analysis between 2 groups were done using edgeR package in R (v4.0.2). Gene set enrichment analysis was performed with clusterProfiler package in R. For human studies, we obtained TPM values from the GTEx project. For multiple-comparison analysis, we used Kruskal-Wallis test with post hoc Dunn’s test with Bonferroni correction.

### Histology.

Mouse tissues were fixed with 4% paraformaldehyde, embedded in paraffin, and sectioned at 5 μm. Sections were stained with H&E or Oil Red O or subjected to immunohistochemical stain. For immunohistochemical stain of pancreas, anti-insulin antibody (Santa Cruz Biotechnology, 8033, 1:1,000) and DAB substrate kit (Vector Laboratories, SK-4100) were used. For immunohistochemical stain of brains, frozen sections were cut at a thickness of 20 μm using a cryostat (Leica Microsystems, CM3050S), and anti–phospho-STAT3 antibody (Cell Signaling Technologies, 9145, 1:200), anti–phospho-S6 ribosomal protein antibody (Cell Signaling Technologies, 4858, 1:500), and Alexa Fluor 488 anti–rabbit IgG (Invitrogen, A11008, 1:1,000) were used. Sections were rinsed and mounted on glass slides using mounting medium and captured using a fluorescence microscope (Keyence, BZ-X810).

### Data availability.

The bioinformatic data used for the analyses described in this manuscript were obtained from the NCBI GEO database (GSE148641) or the GTEx project (dbGaP accession number phs000424.v8.p2).

### Statistics.

Unless otherwise specified, all results are expressed as mean ± SEM; box plots show the interquartile range (box), median (line), and minimum and maximum (whiskers). Statistical significance was determined by the 2-tailed Student’s *t* test or the 1-way or 2-way ANOVA with Bonferroni post hoc test.

### Study approval.

All animal experiments were approved by the Committee for the Ethical Use of Experimental Animals at Gifu Pharmaceutical University, Gifu University, and Kanazawa University.

## Author contributions

GP, KF, and EH conceived and designed the study. GP, KF, TH, YM, Y Inaba, T Tatsuno, TY, KT, SI, and TI performed experiments. KK, YK, Y Ishigaki, MM, T Tanaka, KO, HO, SS, YBS, HI, and HL discussed the results, conceived some experiments, and provided critical reagents. GP, KF, and EH wrote the manuscript. All authors commented on the manuscript and approved the manuscript. GP and KF contributed equally.

## Supplementary Material

Supplemental data

Supplemental tables 1-12

## Version 1 03/02/2023

In-Press Preview

## Version 2 04/10/2023

Electronic publication
