# Supplementary material for: l-Type amino acid transporter 1 in hypothalamic neurons in mice maintains energy and bone homeostasis
Source: JCI Insight. 2023 Apr 10;8(7):e154925. doi: 10.1172/jci.insight.154925 (PMC10132163; doi:10.1172/jci.insight.154925)
Supplement: Supplemental data [file jciinsight-8-154925-s072.pdf]

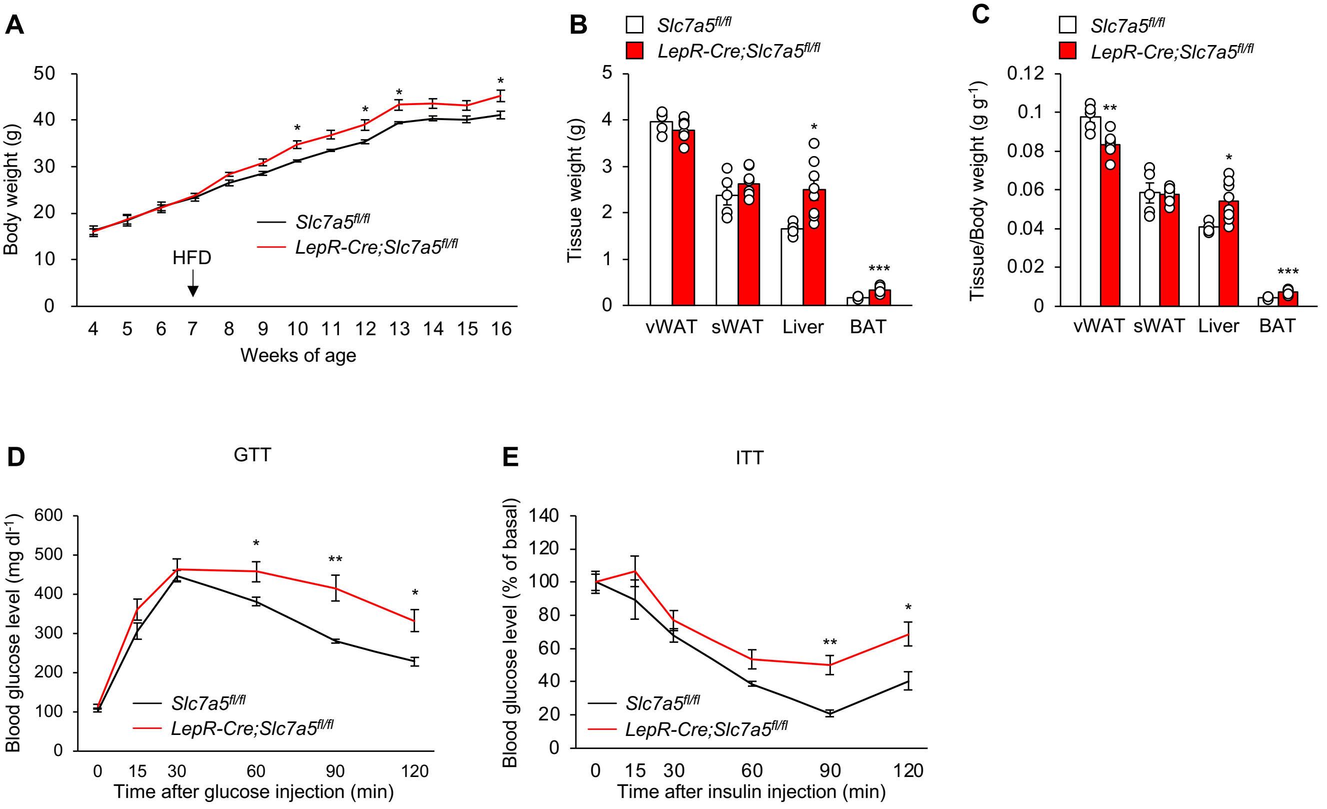

Supplemental Figure. 1 *LepR-Cre* mice does not display any abnormal energy homeostasis.

(**A**) Weekly body weight is shown for *LepR-Cre* mice and control mice fed a NC (*n* = 6, two-way ANOVA with Bonferroni *post hoc* test). (**B**) GTTs were performed in *LepR-Cre* mice and control mice after a 6 hr fast at 16 weeks of age (*n* = 6, two-way ANOVA with Bonferroni *post hoc* test). (**C**) ITTs were performed in *LepR-Cre* mice and control mice after a 6 hr fast at 16 weeks of age (*n* = 6, two-way ANOVA with Bonferroni *post hoc* test). (**D** and **E**) Adipose tissue weights (**D**) and adipose tissue weights normalized to body weight **(E)** are shown for *LepR-Cre* mice and control mice at 16 weeks of age (*n* = 6, two-tailed Student's *t*-test). All of the mice used in this study were male.

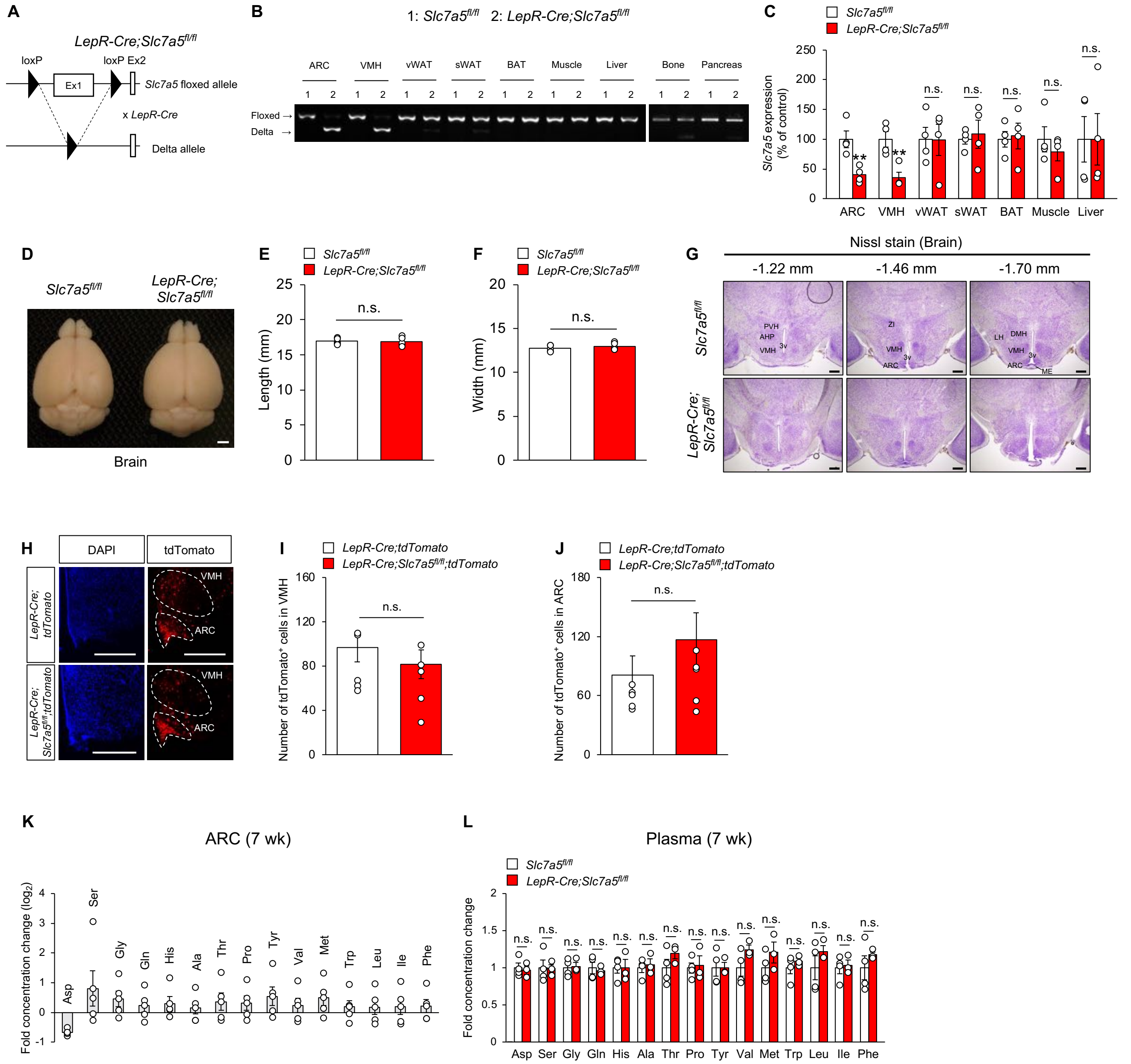

Supplemental Figure. 2 Deletion efficiency and specificity, brain morphology and amino acid levels in *LepR-Cre;Slc7a5^fl/fl^* mice.

(**A**) Schematic diagram of generating LepR-expressing cell-specific *Slc7a5* knockout mice. (**B**) Deletion efficiency of *Slc7a5* in each region of the hypothalamus and peripheral tissues of *LepR-Cre;Slc7a5^fl/fl^* mice at the genomic DNA level (floxed: 2004 bp, delta: 253 bp). (**C**) mRNA levels in each region of the hypothalamus and peripheral tissues of *LepR-Cre;Slc7a5^fl/fl^* mice. (n = 4, **P < 0.01, two-tailed Student's t-test). (**D-F**) Representative picture (**D**), length (**E**) and width (**F**) of brains are shown for *LepR-Cre;Slc7a5^fl/fl^* mice and control mice at 7 weeks of age (n = 5, two-tailed Student's t-test). Scale bar, 1 mm. (**G**) Nissl stain was performed on the brains of *LepR-Cre;Slc7a5^fl/fl^* mice at 7 weeks of age. Scale bar, 100 µm. (**H-J**) Representative images of VMH and ARC (**H**) and the number of tdTomato$^+$ neurons in the VMH (**I**) and ARC (**J**) of *LepR-Cre;Slc7a5^fl/fl^;tdTomato* mice at 7 weeks of age (n = 7, two-tailed Student's t-test). Scale bar, 500 µm. (**K**) Log$_2$ ratio of the amino acid levels in ARC between *LepR-Cre;Slc7a5^fl/fl^* mice and control mice at 7 weeks of age (n = 5, two-tailed Student's t-test). (**L**) Amino acid levels in plasma of *LepR-Cre;Slc7a5^fl/fl^* mice at 7 weeks of age (n = 3 or 4, two-tailed Student's t-test). All of the mice used in this study were male.

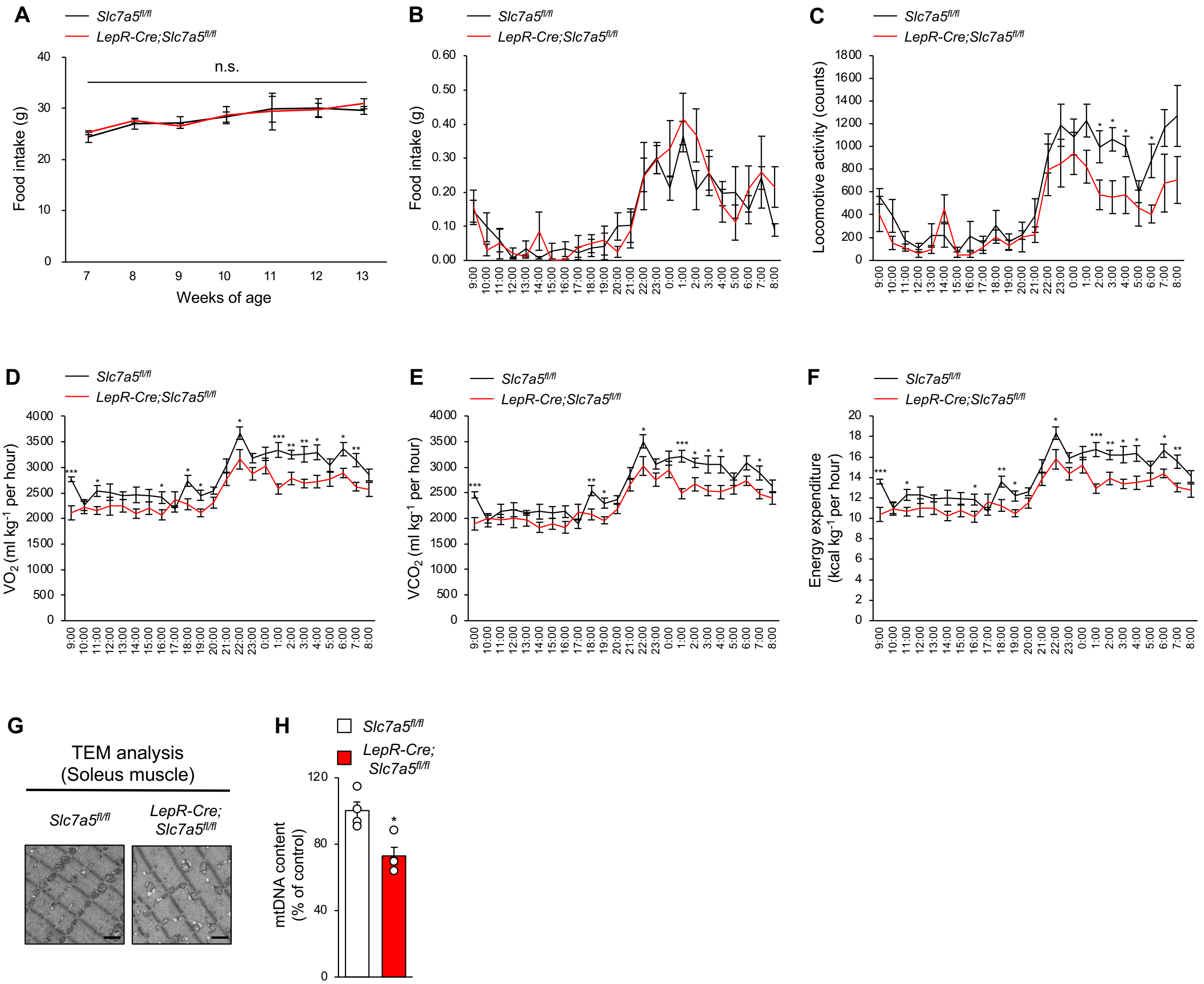

Supplemental Figure. 3 Metabolic parameters and muscle mitochondrial morphology in *LepR-Cre;Slc7a5$^{fl/fl}$* mice.

(**A**) Weekly food intake is shown for *LepR-Cre;Slc7a5$^{fl/fl}$* mice and control mice fed a NC (n = 7 or 9, two-way ANOVA with Bonferroni *post hoc* test), (**B**-**F**) Temporal changes of food intake (**B**), locomotor activity (**C**), O_2 consumption (**D**), CO_2 production (**E**) and energy expenditure (**F**) were measured in singly housed *LepR-Cre;Slc7a5$^{fl/fl}$* mice and control mice at 22-24 weeks of age (n = 7 or 8, *$P < 0.05$, **$P < 0.01$, ***$P < 0.001$, two-way ANOVA with Bonferroni *post hoc* test). (**G**) TEM analysis was performed on the soleus muscle of *LepR-Cre;Slc7a5$^{fl/fl}$* mice and control mice at 22-24 weeks of age. Scale bar, 10 μm. (**H**) mtDNA content of soleus muscle was measured in *LepR-Cre;Slc7a5$^{fl/fl}$* mice and control mice at 22-24 weeks of age (n = 4, *$P < 0.05$, two-tailed Student's t-test). All of the mice used in this study were male.

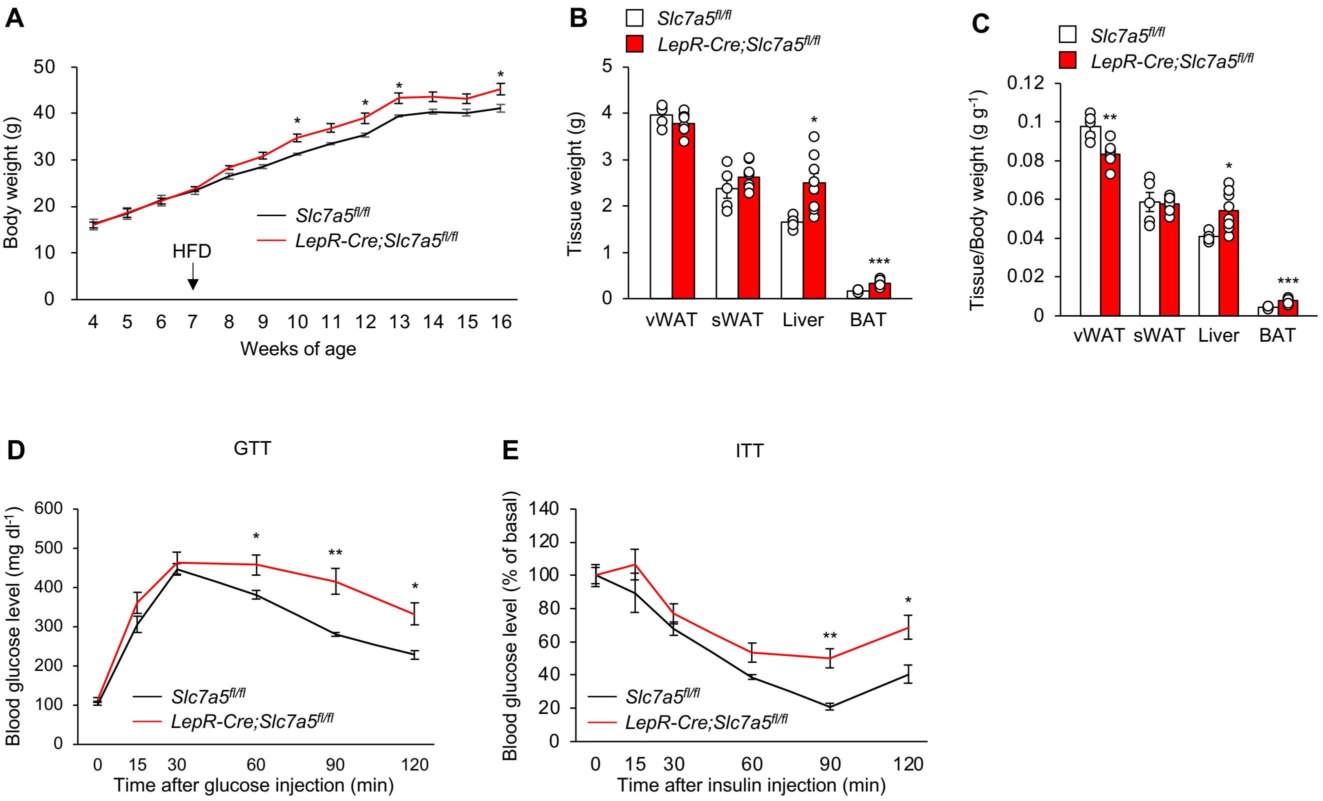

Supplemental Figure. 4 HFD-induced obesity is exacerbated in *LepR-Cre;Slc7a5$^{fl/fl}$* mice.

(**A**) Weekly body weight is shown for *LepR-Cre;Slc7a5$^{fl/fl}$* mice and control mice fed a HFD (n = 5 or 8, *$P < 0.05$, two-way ANOVA with Bonferroni *post hoc* test). (**B** and **C**) Adipose tissue weights (**B**) and adipose tissue weights normalized to body weight **(C)** are shown for *LepR-Cre;Slc7a5$^{fl/fl}$* mice and control mice at 16 weeks of age (n = 5 or 8, *$P < 0.05$, **$P < 0.01$, ***$P < 0.001$, two-tailed Student's t-test). (**D**) GTTs were performed in *LepR-Cre;Slc7a5$^{fl/fl}$* mice and control mice after a 6 hr fast at 16 weeks of age (n = 6, *$P < 0.05$, **$P < 0.01$). (**E**) ITTs were performed in *LepR-Cre;Slc7a5$^{fl/fl}$* mice and control mice after a 6 hr fast at 16 weeks of age (n = 6, *$P < 0.05$, **$P < 0.01$, two-way ANOVA with Bonferroni *post hoc* test). All of the mice used in this study were male.

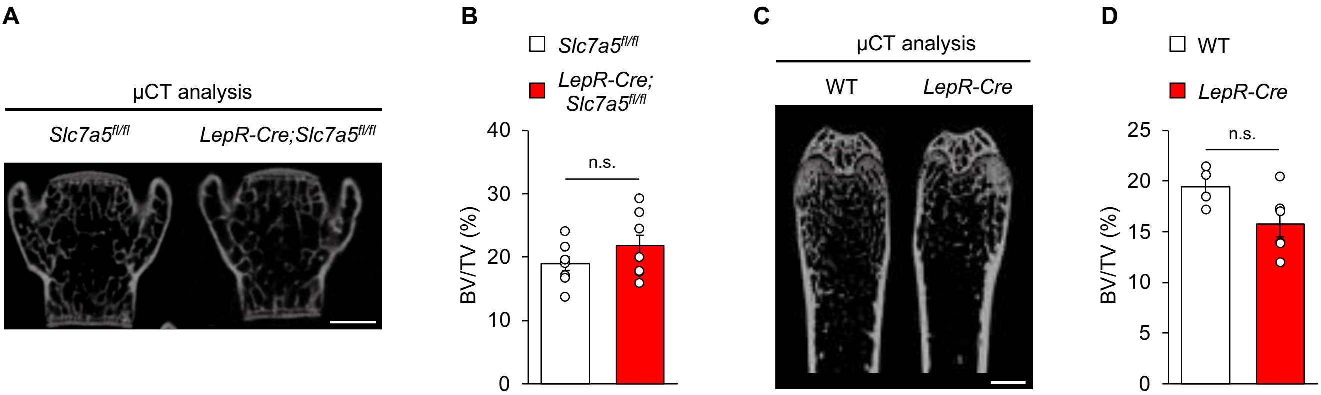

Supplemental Figure. 5 Analysis of bone volume in vertebrae from *LepR-Cre;Slc7a5$^{fl/fl}$* mice and femurs from *LepR-Cre* mice.

(**A**) µCT analysis, scale bar, 1 mm and (**B**) BV/TV ratio as determined by µCT of vertebrae from *LepR-Cre;Slc7a5$^{fl/fl}$* mice at 14 weeks of age (*n* = 7 or 8, two-tailed Student's *t*-test). (**C**) µCT analysis, scale bar, 1 mm, and (**D**) BV/TV ratio as determined by µCT of femurs from *LepR-Cre* mice and control mice at 12-16 weeks of age (*n* = 4 or 6, two-tailed Student's *t*-test). All of the mice used in this study were male.

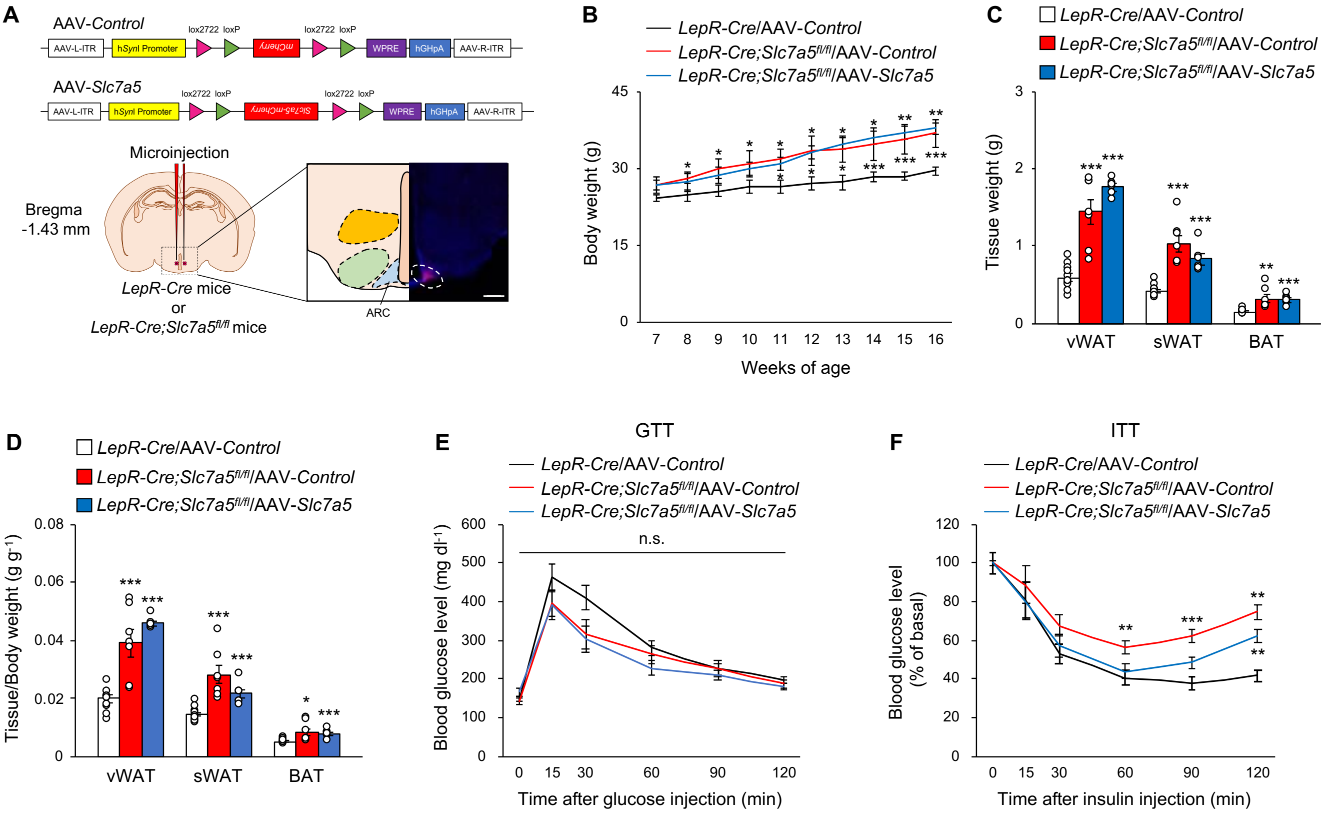

Supplemental Figure. 6 LepR-expressing ARC neurons do not contribute to LAT1-dependent regulation of systemic energy and bone homeostasis.

(**A**) Schematic diagram of the bilateral viral microinjection into the ARC, and representative validation image of mCherry expression in the ARC. Scale bar, 500 μm. (**B**) Weekly body weight after injection of AAV-*Control* or AAV-*Slc7a5* into the ARC is shown for *LepR-Cre;Slc7a5*$^{fl/fl}$ mice and control mice (n = 6 to 11, *P < 0.05: versus *LepR-Cre*/AAV-*Control*, two-way ANOVA with Bonferroni *post hoc* test). (**C** and **D**) Adipose tissue weights (**C**) and adipose tissue weights normalized to body weight (**D**) are shown for *LepR-Cre;Slc7a5*$^{fl/fl}$ mice and control mice injected with AAV-*Control* or AAV-*Slc7a5* into the ARC at 16 weeks of age (n = 6 to 11, *P < 0.05, **P < 0.01, ***P < 0.001, two-tailed Student's t-test with Bonferroni correction). (**E**) GTTs were performed in *LepR-Cre;Slc7a5*$^{fl/fl}$ mice and control mice after a 6 hr fast at 16 weeks of age (n = 6 to 11, two-way ANOVA with Bonferroni *post hoc* test). (**F**) ITTs were performed in *LepR-Cre;Slc7a5*$^{fl/fl}$ mice and control mice injected with AAV-*Control* or AAV-*Slc7a5* into the ARC after a 6 hr fast at 16 weeks of age (n = 6 to 11, **P < 0.01, ***P < 0.001: versus *LepR-Cre*/AAV-*Control*, two-way ANOVA with Bonferroni *post hoc* test). All of the mice used in this study were male.

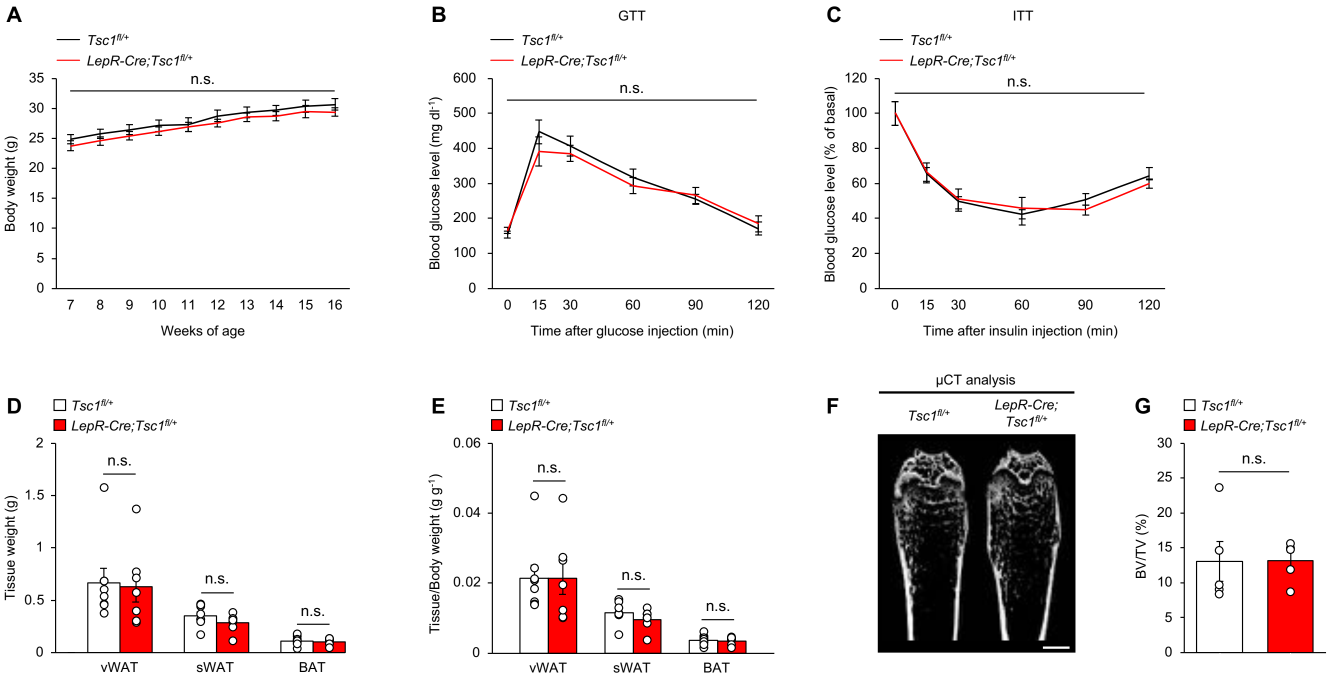

Supplemental Figure. 7 ***LepR-Cre;Tsc1***$^{fl/+}$ **mice does not display any abnormal energy homeostasis.**

(**A**) Weekly body weight is shown for *LepR-Cre;Tsc1*$^{fl/+}$ mice and control mice fed a NC (n = 7 or 8, two-way ANOVA with Bonferroni *post hoc* test). (**B**) GTTs were performed in *LepR-Cre;Tsc1*$^{fl/+}$ mice and control mice after a 6 hr fast at 16 weeks of age (n = 7 or 8, two-way ANOVA with Bonferroni *post hoc* test). (**C**) ITTs were performed in *LepR-Cre;Tsc1*$^{fl/+}$ mice and control mice after a 6 hr fast at 16 weeks of age (n = 7 or 8, two-way ANOVA with Bonferroni *post hoc* test). (**D** and **E**) Adipose tissue weights (**D**) and adipose tissue weights normalized to body weight (**E**) are shown for *LepR-Cre;Tsc1*$^{fl/+}$ mice and control mice at 16 weeks of age (n = 7 or 8, two-tailed Student's t-test). (**F**) µCT analysis, scale bar, 1 mm, and (**G**) BV/TV ratio as determined by µCT of femurs from *LepR-Cre;Tsc1*$^{fl/+}$ mice and control mice at 12-16 weeks of age (n = 5, two-tailed Student's t-test). All of the mice used in this study were male.
