# Supplementary material for: l-Type amino acid transporter 1 in hypothalamic neurons in mice maintains energy and bone homeostasis
Source: JCI Insight. 2023 Apr 10;8(7):e154925. doi: 10.1172/jci.insight.154925 (PMC10132163; doi:10.1172/jci.insight.154925)
Supplement: Supplemental tables 1-12 [file jciinsight-8-154925-s073.pdf]

Supplemental Table

Supplemental Table 1. Bone parameters measured by µCT in *LepR-Cre;Slc7a5*$^{fl/fl}$ mice.

	Slc7a5$^{fl/fl}$	*LepR-Cre;Slc7a5*$^{fl/fl}$
**TV (mm^3)**	2.05 ± 0.06	2.17 ± 0.08
**BV (mm^3)**	0.38 ± 0.02	0.49 ± 0.04 *
**BS (mm^2)**	19.14 ± 0.74	23.11 ± 1.27 *
**BS/BV (mm^{-1})**	51.41 ± 1.6	48.36 ± 1.44
**Tb.Th (µm)**	57.39 ± 1.88	60.97 ± 1.49
**Tb.N (mm^{-1})**	2.29 ± 0.3	2.31 ± 0.09
**Tb.Sp (µm)**	142.24 ± 3.44	127.75 ± 3.39 **
**Tb.Spac (µm)**	199.63 ± 4.4	188.72 ± 3.56
**Trabecular BMD (mg cm^{-3})**	618.59 ± 11.56	624.38 ± 9.72
**Trabecular BMC (mg)**	0.24 ± 0.02	0.31 ± 0.02 *
**Trabecular BMC/TV (mg cm^{-3})**	114.57 ± 6.9	139.58 ± 8.39 *
**Cortical BMD (mg cm^{-3})**	974.66 ± 26.05	977.18 ± 21.43
**Cortical BMC (mg)**	1.15 ± 0.05	1.17 ± 0.03
**Cortical BV (mm^3)**	1.18 ± 0.03	1.2 ± 0.03

Data are represented as mean ± SE.

*$P < 0.05$, **$P < 0.01$ vs *Slc7a5*$^{fl/fl}$

Supplemental Table 2. Bone histomorphometric parameters in *LepR-Cre;Slc7a5*$^{fl/fl}$ mice.

	Slc7a5$^{fl/fl}$	*LepR-Cre;Slc7a5*$^{fl/fl}$
**N.Ob/B.Pm**	25.71 ± 3.62	27.34 ± 0.99
**Ob.S/B.S**	18.65 ± 3.18	18.06 ± 1.43
**N.Ob/Ob.Pm**	126.84 ± 2.91	143.01 ± 6.47

Data are represented as mean ± SE.

Supplemental Table 3. Bone parameters measured by μCT in *LepR-Cre;Slc7a5*$^{fl/fl}$ mice administrated with isoproterenol.

	Slc7a5$^{fl/fl}$	***LepR-Cre;Slc7a5***$^{fl/fl}$	***LepR-Cre;Slc7a5***$^{fl/fl}$
	**PBS**	**PBS**	**Isoproterenol**
**TV (mm^3)**	1.96 ± 0.10	2.08 ± 0.09	1.93 ± 0.10
**BV (mm^3)**	0.3 ± 0.03	0.41 ± 0.03 *	0.28 ± 0.05 $^{\#}$
**BS (mm^2)**	13.36 ± 1.20	19.18 ± 1.12 **	12.55 ± 1.69 $^{\#\#}$
**BS/BV (mm^{-1})**	45.03 ± 1.65	46.70 ± 1.55	47.27 ± 2.77
**Tb.Th (μm)**	44.92 ± 1.55	43.29 ± 1.55	43.46 ± 2.19
**Tb.N (mm^{-1})**	3.37 ± 0.20	4.62 ± 0.18 ***	3.20 ± 0.33 $^{\#\#}$
**Tb.Sp (μm)**	263.65 ± 21.93	176.54 ± 9.33 **	303.77 ± 41.31 $^{\#}$
**Tb.Spac (μm)**	308.57 ± 21.81	219.83 ± 9.40 **	347.23 ± 39.26 $^{\#\#}$
**Trabecular BMD (mg cm^{-3})**	568.9 ± 17.2	524.59 ± 20.56	561.36 ± 19.39
**Trabecular BMC (mg)**	0.17 ± 0.02	0.22 ± 0.02	0.16 ± 0.03
**Trabecular BMC/TV (mg cm^{-3})**	85.28 ± 6.80	104.76 ± 7.83	82.82 ± 12.45
**Cortical BMD (mg cm^{-3})**	978.16 ± 14.74	955.37 ± 22.17	966.06 ± 15.92
**Cortical BMC (mg)**	1.15 ± 0.06	1.10 ± 0.06	1.11 ± 0.06
**Cortical BV (mm^3)**	1.17 ± 0.06	1.15 ± 0.04	1.15 ± 0.05

Data are represented as mean ± SE.

*$P < 0.05$, **$P < 0.01$, ***$P < 0.001$ vs *Slc7a5*$^{fl/fl}$/PBS

$^{\#}P < 0.05$, $^{\#\#}P < 0.01$ vs *LepR-Cre;Slc7a5*$^{fl/fl}$/PBS

Supplemental Table 4. Bone parameters measured by µCT in *LepR-Cre* mice.

	**WT**	***LepR-Cre***
**TV (mm^3)**	1.93 ± 0.05	2.05 ± 0.05
**BV (mm^3)**	0.37 ± 0.01	0.32 ± 0.03
**BS (mm^2)**	17.73 ± 1.06	15.67 ± 0.88
**BS/BV (mm^{-1})**	47.28 ± 2.15	49.35 ± 2.53
**BV/TV (%)**	19.44 ± 0.97	15.76 ± 1.25
**Tb.Th (µm)**	42.59 ± 2.10	41.06 ± 2.11
**Tb.N (mm^{-1})**	4.60 ± 0.33	3.83 ± 0.18
**Tb.Sp (µm)**	178.36 ± 15.10	223.41 ± 13.65
**Tb.Spac (µm)**	220.95 ± 16.67	264.47 ± 13.01
**Trabecular BMD (mg cm^{-3})**	529.60 ± 30.12	645.38 ± 67.34
**Trabecular BMC (mg)**	0.20 ± 0.01	0.20 ± 0.02
**Trabecular BMC/TV (mg cm^{-3})**	101.65 ± 5.22	98.47 ± 7.90
**Cortical BMD (mg cm^{-3})**	962.30 ± 35.74	1132.00 ± 103.07
**Cortical BMC (mg)**	1.18 ± 0.14	1.21 ± 0.10
**Cortical BV (mm^3)**	1.22 ± 0.10	1.08 ± 0.06

Data are represented as mean ± SE.

Supplemental Table 5. Bone parameters measured by μCT in *LepR-Cre;Slc7a5$^{fl/fl}$*/AAV-*Slc7a5* mice.

	LepR-Cre	***LepR-Cre; Slc7a5$^{fl/fl}$***	***LepR-Cre***	***LepR-Cre; Slc7a5$^{fl/fl}$***
	**AAV-*Control***	**AAV-*Control***	**AAV-*Slc7a5***	**AAV-*Slc7a5***
**TV (mm^3)**	2.33 ± 0.07	2.28 ± 0.08	2.43 ± 0.08	2.1 ± 0.07 †
**BV (mm^3)**	0.38 ± 0.03	0.5 ± 0.02	0.36 ± 0.02	0.3 ± 0.05 $^{\#\#}$
**BS (mm^2)**	20.44 ± 0.95	24.48 ± 0.79	19.73 ± 0.8	16.29 ± 1.69 $^{\#\#\#}$
**BS/BV (mm^{-1})**	55.08 ± 1.94	49.35 ± 2.18	55.31 ± 0.79	56.63 ± 3.29
**Tb.Th (μm)**	36.84 ± 1.51	41.01 ± 1.81	36.19 ± 0.52	36.05 ± 2.12
**Tb.N (mm^{-1})**	4.38 ± 0.12	5.37 ± 0.13 **	4.06 ± 0.1	3.85 ± 0.34 $^{\#\#\#}$
**Tb.Sp (μm)**	193.32 ± 6.85	145.77 ± 5.34	210.87 ± 6.85	238.57 ± 30.23 $^{\#\#}$
**Tb.Spac (μm)**	230.15 ± 6.16	186.77 ± 4.17	247.06 ± 6.6	274.61 ± 28.47 $^{\#\#}$
**Trabecular BMD (mg cm^{-3})**	614.55 ± 11.65	631.91 ± 19.86	599.08 ± 6.44	592.1 ± 14.1
**Trabecular BMC (mg)**	0.24 ± 0.02	0.32 ± 0.02	0.22 ± 0.01	0.19 ± 0.03 $^{\#\#}$
**Trabecular BMC/TV (mg cm^{-3})**	100.57 ± 7.42	140.36 ± 12.62 *	88.77 ± 3.74	85.94 ± 13.35 $^{\#\#}$
**Cortical BMD (mg cm^{-3})**	1008.19 ± 11.4	1002.66 ± 11.86	1027.58 ± 5.92	1041.4 ± 12.86
**Cortical BMC (mg)**	1.11 ± 0.04	1.25 ± 0.05	1.11 ± 0.02	1.16 ± 0.04
**Cortical BV (mm^3)**	1.1 ± 0.03	1.25 ± 0.04 *	1.08 ± 0.02	1.12 ± 0.04

Data are represented as mean ± SE.

*$P < 0.05$, **$P < 0.01$ vs *LepR-Cre*/AAV-*Control*

$^{\dagger}P < 0.05$ vs *LepR-Cre*/AAV-*Slc7a5*

$^{\#\#}P < 0.01$, $^{\#\#\#}P < 0.001$ vs *LepR-Cre;Slc7a5$^{fl/fl}$*/AAV-*Control*

Supplemental Table 6. Bone parameters measured by μCT in *LepR-Cre;Slc7a5*$^{fl/fl}$*;Tsc1*$^{fl/+}$ mice.

	Slc7a5$^{fl/fl}$	*LepR-Cre;Slc7a5*$^{fl/fl}$	*LepR-Cre; Slc7a5*$^{fl/fl}$*;Tsc1*$^{fl/+}$
**TV (mm^3)**	2.01 ± 0.04	2.35 ± 0.08 **	1.98 ± 0.12 #
**BV (mm^3)**	0.36 ± 0.02	0.58 ± 0.04 ***	0.37 ± 0.04 ##
**BS (mm^2)**	18.93 ± 0.72	26.58 ± 1.54 ***	19.31 ± 2.23 ##
**BS/BV (mm^{-1})**	52.79 ± 1.4	46.37 ± 1.1 *	52.78 ± 0.69
**Tb.Th (μm)**	18.04 ± 0.88	24.36 ± 1 ***	18.41 ± 1.21 #
**Tb.N (mm^{-1})**	57.35 ± 0.95	61.05 ± 0.95	57.4 ± 1.92
**Tb.Sp (μm)**	2.01 ± 0.06	2.51 ± 0.09 ***	2.01 ± 0.13 ##
**Tb.Spac (μm)**	139.03 ± 2.52	123.01 ± 3.84 **	134.31 ± 6.31
**Trabecular BMD (mg cm^{-3})**	196.38 ± 2.49	184.06 ± 4.26	191.71 ± 8.17
**Trabecular BMC (mg)**	599.56 ± 6.83	633.75 ± 10.42 *	596.3 ± 8.37
**Trabecular BMC/TV (mg cm^{-3})**	0.22 ± 0.01	0.37 ± 0.03 ***	0.22 ± 0.02 ##
**Cortical BMD (mg cm^{-3})**	108.61 ± 5.97	153.9 ± 8.1 ***	109.5 ± 6.11 #
**Cortical BMC (mg)**	981.61 ± 22.62	977.88 ± 25.29	1023.77 ± 17.98
**Cortical BV (mm^3)**	1.1 ± 0.04	1.22 ± 0.06	1.11 ± 0.07
**Cortical BV (mm^3)**	1.12 ± 0.03	1.24 ± 0.03 *	1.08 ± 0.06

Data are represented as mean ± SE.

*$P < 0.05$, **$P < 0.01$, ***$P < 0.001$ vs *Slc7a5*$^{fl/fl}$

$^{\#}P < 0.05$, $^{\#\#}P < 0.01$ vs *LepR-Cre;Slc7a5*$^{fl/fl}$

Supplemental Table 7. Bone parameters measured by µCT in *LepR-Cre;Tsc1$^{fl/+}$* mice.

	Tsc1$^{fl/+}$	***LepR-Cre;Tsc1$^{fl/+}$***
**TV (mm^3)**	1.98 ± 0.13	2.13 ± 0.09
**BV (mm^3)**	0.27 ± 0.08	0.28 ± 0.03
**BS (mm^2)**	15.61 ± 2.92	16.93 ± 1.88
**BS/BV (mm^{-1})**	62.90 ± 4.97	60.45 ± 2.04
**Tb.Th (µm)**	13.05 ± 2.86	13.13 ± 1.28
**Tb.N (mm^{-1})**	32.78 ± 3.12	33.23 ± 1.08
**Tb.Sp (µm)**	3.83 ± 0.47	3.94 ± 0.33
**Tb.Spac (µm)**	242.90 ± 32.84	229.93 ± 28.18
**Trabecular BMD (mg cm^{-3})**	275.68 ± 30.38	263.16 ± 27.87
**Trabecular BMC (mg)**	739.28 ± 40.88	758.48 ± 32.21
**Trabecular BMC/TV (mg cm^{-3})**	0.21 ± 0.07	0.22 ± 0.03
**Cortical BMD (mg cm^{-3})**	100.36 ± 27.98	100.2 ± 11.73
**Cortical BMC (mg)**	1490.18 ± 66.79	1479.64 ± 48.71
**Cortical BV (mm^3)**	1.44 ± 0.14	1.43 ± 0.07
**Cortical BV (mm^3)**	0.97 ± 0.10	0.96 ± 0.02

Data are represented as mean ± SE.

Supplemental Table 8. List of primers used for genotyping.

Gene	Forward (5’-3’)	Reverse (5’-3’)
Slc7a5 flox	GGCTCCTGGACTTATCTTGACCAAT	AGATAATGTGGTCACACATCTGGAAG
Tsc1 flox	AGGAGGCCTCTTCTGCTACCACTTTT GATG	GAAGGCAGCTCCGACCATGAAGTGCT GTGT
LepR-Cre	ATGTCCAATTTACTGACCGTACA	CGCATAACCAGTGAAACAGCATT
Rosa26-tdTomato	GGCATTAAAGCAGCGTATCC	CTGTTCCTGTACGGCATGG

Supplemental Table 9. List of primers used for deletion PCR.

Gene	Forward (5’-3’)	Reverse (5’-3’)
Slc7a5	GGCTCCTGGACTTATCTTGACCAATG	GTGGTGCTTTGCTGAAGGCAGGG

Supplemental Table 10. List of primers used for real-time PCR.

Gene	Forward (5’-3’)	Reverse (5’-3’)
Adrb3	TGCGCACCTTAGGTCTCATTATGG	AAACTCCGCTGGGAACTAGAGAGG
Cidea	ATCACAACTGGCCTGGTTACG	TACTACCCGGTGTCCATTTCT
Cox5b	GCTGCATCTGTGAAGAGGACAAC	CAGCTTGTAATGGGTTCCACAGT
Cox7a1	CAGCGTCATGGTCAGTCTGT	AGAAAACCGTGTGGCAGAGA
Cox8b	GAACCATGAAGCCAACGACT	GCGAAGTTCACAGTGGTTCC
Cyc1	GCTACCCATGGTCTCATCGT	CATCATCATTAGGGCCATCC
Dio2	GGTGGTCAACTTTGGTTCAGCC	AAGTCAGCCACCGAGGAGAACT
Gapdh	AGGTCGGTGTGAACGGATTTG	TGTAGACCATGTAGTTGAGGTCA
Il6	CACCAAGAACGATAGTCAATTCCA	TCACCAGCATCAGTCCCAAG
Nrf1	CAACAGGGAAGAAACGGAAA	GCACCACATTCTCCAAAGGT
Nrf2	AGGTTGCCCACATTCCCAAACAAG	TTGCTCCATGTCCTGCTCTATGCT
Pgc1a	GAATCAAGCCACTACAGACACCG	CATCCCTCTTGAGCCTTTCGTG
Tfam	GTCCATAGGCACCGTATTGC	CCCATGCTGGAAAAACACTT
Ucp1	TACCAAGCTGTGCGATGTCC	GCACACAAACATGATGACGTTCC

Supplemental Table 11. List of primers used for real-time PCR to quantify mtDNA.

Gene	Forward (5’-3’)	Reverse (5’-3’)
mtDNA	CCGCAAGGGAAAGATGAAAGAC	TCGTTTGGTTTCGGGGTTTC
Nuclear DNA	GCCAGCCTCTCCTGATTTTAGTGT	GGGAACACAAAAGACCTCTTCTGG
